# Challenges in the development of treatment guidelines for bipolar disorder

**DOI:** 10.3389/fpsyt.2025.1564004

**Published:** 2025-06-10

**Authors:** Konstantinos N. Fountoulakis, Nikolaos K. Fountoulakis, Diomidis Antoniadis

**Affiliations:** ^1^ 3rd Department of Psychiatry, School of Medicine, Aristotle University of Thessaloniki, Thessaloniki, Greece; ^2^ Society for Neurosciences and Rehabilitation (E.N.A.), Thessaloniki, Greece; ^3^ Consultant, NHS, Thessaloniki, Greece

**Keywords:** bipolar disorder, treatment guidelines, treatment algorithms, anticonvulsants, antidepressants, antipsychotics, evidence-based, lithium

## Abstract

Bipolar disorder (BD) is such a complex mental disorder, that even the development of true, reliable, and valid treatment guidelines seems to be a goal almost impossible to achieve. The challenges include the complexity and uniqueness of the clinical picture and the therapeutical options available, special issues including gender, pregnancy, and the different views of therapists and patients. An additional issue is the method for the development of the guidelines, with systematic reviews of the hard evidence to constitute the most recent trend. The grading of the literature findings could be crucial for the whole process, as it is often ‘contaminated’ by expert opinion. Unfortunately, in the literature, BD is treated as a fragmented condition and each fragment is studied separately as if it were independent. This, in combination with incomplete reporting of the findings, makes the synthesis of the landscape almost impossible and the development of a comprehensive single algorithm for the continuous treatment of BD, extremely difficult. Overall, developing treatment guidelines for BD constitutes a great challenge. This task demands an exhaustive review of the existing literature, searching for unpublished data and digging deep into them to comprehend their nature. It also needs to manage to synthesize the fragmented research picture that refers to isolated faces of the disorder, into a comprehensive network of decision-making that will incorporate the knowledge of the past with decisions for the present by having the mind in the future (the three-fingers rule).

## 1 Introduction

Bipolar disorder (BD) is an incredibly complex condition—so much so that even among mental health disorders, its intricacies stand out. A wide range of factors contribute to its development and course, including biological, psychological, social, and even environmental influences (1–3). Given this complexity, precise treatment guidelines are crucial for clinicians. Over the past few decades, these guidelines have become an increasingly vital part of modern medicine. This is important, especially as the sheer volume of research—often intricate and sometimes contradictory—makes it harder to translate findings into everyday clinical practice.

These guidelines serve multiple purposes: they help clinicians and policymakers make informed decisions about patient care, establish standards for healthcare professionals, and highlight key areas where more research is needed. While they are primarily based on existing scientific evidence, in cases where research is lacking, expert consensus helps fill the gaps (4–6). A list of the most important contemporary guidelines is shown in **Table 1**.

**Table 1:** List of the most important contemporary guidelines for Bipolar disorder.

1.	BAP (7)
2.	CANMAT/ISBD (8)
3.	CINP (9–11)
4.	Korean (12)
5.	NICE (13)
6.	RANZCP (14)
7.	WFSBP (15–18)

That said, as we will explore further, the current body of knowledge on BD treatment and management is so vast and complex that developing truly comprehensive and universally reliable treatment guidelines remains an incredibly difficult, if not nearly impossible, challenge (19).

## 2 Challenges stemming from the clinical picture

The goal of treatment for BD is to relieve clinical symptoms, reduce suffering, and help individuals regain their ability to function in daily life. What makes BD particularly challenging compared to other mental health conditions is its complex and unpredictable nature. It presents with distinct episodes—some completely different from one another—that can appear independently or share overlapping features. Unlike schizophrenia, which tends to follow a more stable long-term course, BD is marked by unpredictable fluctuations in symptoms and levels of disability.

A detailed breakdown of the different facets and syndromes that make up BD can be found in **Table 2**. The most pressing clinical challenges—which in turn complicate treatment efforts and the development of clear treatment guidelines—are outlined below.

**Figure 1:**
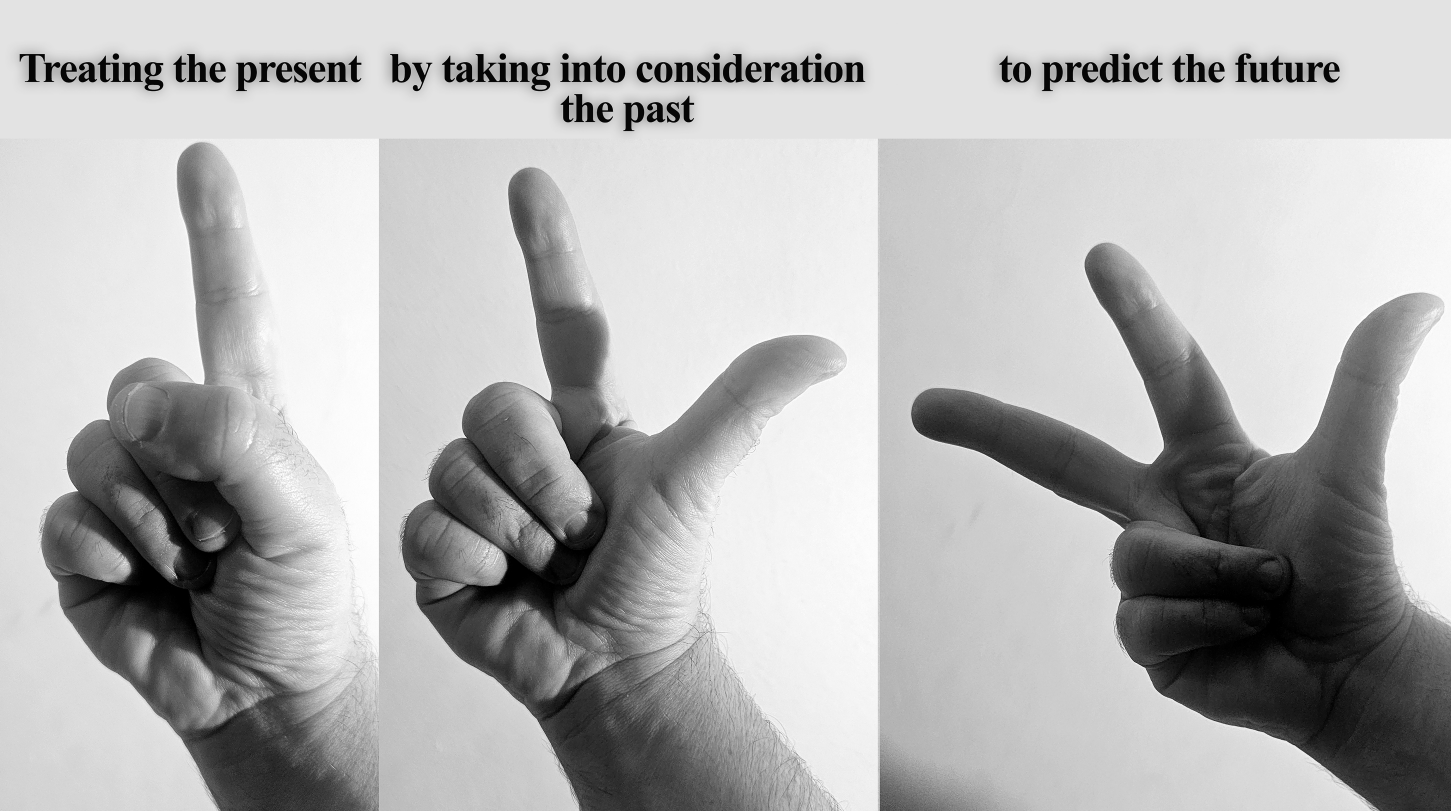
The three-fingers rule: treating the present by taking into consideration the past to predict the future.

- **Difficulty in making early correct diagnosis** One of the biggest challenges in managing BD is accurately diagnosing it early on (20–23). In fact, up to 70% of patients experience a depressive episode first, leading to a misdiagnosis of unipolar depression and, as a result, inappropriate treatment with antidepressant monotherapy (20, 22, 24–26). In some cases, manic symptoms may not appear until 20 years after the initial depressive episode, further prolonging the delay in receiving the correct diagnosis (27, 28). Treatment guidelines should focus on strategies to navigate this “grey zone” of diagnostic uncertainty and help clinicians identify BD as early as possible.
- **Suicidal thoughts** are another major concern, affecting 78.6% of BD patients at some point in their lives (29). BD is also one of the psychiatric conditions with the highest risk of suicide attempts and completed suicides (30–34). Suicidality is often an acute crisis requiring immediate intervention, yet the most effective long-term treatments, such as lithium and lamotrigine, take time to work, while others, like certain antidepressants, may even be harmful. Because of this, treatment guidelines should establish a structured approach—an algorithmic strategy that carefully balances the need for rapid symptom relief with long-term stability, ensuring that urgent interventions do not come at the cost of worsening the overall prognosis.
- Some individuals with BD consistently experience one type of episode more frequently than the other—a pattern known as **predominant polarity**. Nearly half of BD patients fall into this category, with the majority tending toward the depressive pole (35–52). This distinction is critical for treatment, as it suggests that therapy should be tailored to the dominant symptom pattern. Treatment guidelines must take this into account, encouraging clinicians to approach current symptoms by considering past patterns while also anticipating future episodes (treating the present by taking into consideration the past to predict the future; the three-fingers rule, **Figure 1**).
- BD can also present with **rapid cycling**, a pattern in which mood episodes switch more frequently than usual. Variants include rapid, ultra-rapid, ultra-ultra-rapid, and ultradian cycling, with mood shifts occurring anywhere from months to within a single day. At some point in their illness, anywhere from one-quarter to one-half of BD patients will experience some form of rapid cycling (53–67). Treating these patients is especially challenging because very few medications work well for both poles of BD, and most require time to take effect. As a result, treatment guidelines must find a careful balance—providing swift relief for immediate symptoms without disrupting the long-term stability of the patient.
- Another key factor in treatment planning is the **long-term course of BD**, which varies widely among patients. In most cases (69.6%), BD follows a recurrent episodic pattern with distinct periods of illness and remission. However, in 25% of cases, the disorder takes a more chronic course, with continuous symptoms and little to no remission. A small percentage of patients (5.4%) experience only a single manic episode, while around 5% have chronic mania without depressive episodes (29). Since different long-term patterns require fundamentally different treatment approaches, treatment guidelines must acknowledge this variability and provide flexible strategies that can be adapted to each patient’s unique trajectory.
- **The neurocognitive impairment** in BD affects nearly all areas of thinking and occurs across different phases of the illness. Research has shown that psychosocial functioning is closely linked to processing speed (68, 69), abstract thinking (68), verbal memory, and executive function—even in patients who are in remission (euthymic phase) (70, 71). The severity of cognitive deficits is most pronounced during acute episodes but remains significant, though to a lesser extent, even during euthymia. While BD patients tend to have milder cognitive deficits compared to those with schizophrenia, the pattern of impairment is strikingly similar in both disorders (72, 73).
- A particularly urgent clinical issue in BD is **agitation** and its management. Recently, experts have even published a consensus paper outlining best practices for managing agitation in BD patients, underscoring the need for clear treatment strategies (74).
- Another major challenge in BD treatment is **psychiatric comorbidity**, which complicates diagnosis and management. In BD, having coexisting mental health conditions is the norm rather than the exception (75). Over half to two-thirds of BD patients experience at least one comorbid disorder in their lifetime (76–86), while 42% have two, and 25% have three or more comorbid psychiatric conditions (80). The prevalence of current (cross-sectional) comorbidity is lower, with around one-third of BD patients having at least one additional mental health disorder at any given time (76, 79, 86–88). Comorbidity in BD is associated with a more complex clinical picture, an earlier age of onset (89), and worse long-term outcomes, including higher suicidality and self-harm risk (78, 80, 86, 89, 90), poor treatment adherence (86), and less favorable response to lithium (82, 90). Anxiety disorders, in particular, are highly prevalent in BD, affecting 42–93% of patients at some point in their lifetime and 11–70% at any given time (57, 79, 80, 87, 91–113). Anxiety in BD is also strongly linked to predominant depressive polarity, further influencing treatment decisions and prognosis (96, 114).
- **Alcohol and substance use disorders (SUD)** significantly complicate the course of BD, increasing the risk of legal issues and suicidality (115). Data from the Epidemiological Catchment Area (ECA) study indicate that, at any given time, drug abuse is present in 13% of BD-I and 9% of BD-II patients, while drug dependence is observed in 28% and 12%, respectively (116). SUDs may also serve as either a risk factor or a prognostic marker for the development of BD with psychotic features, with a hazard ratio (HR) of 3 (117). Among BD-I patients, the lifetime prevalence of SUDs is at least 40%, with alcohol and cannabis being the most commonly abused substances, followed by cocaine and opioids (118, 119). Clinical studies suggest that alcohol use disorder affects anywhere from one-third to 75% of BD patients (120–125). According to ECA data, alcohol abuse is found in 15% of BD-I and 18% of BD-II patients, while alcohol dependence is present in 31% and 21%, respectively (116). Other substance use rates in BD patients include cannabis use disorder (5–65%) (F. 126, 127), gambling disorder (6–20%) (128, 129), cocaine abuse or dependence (3–7%) (K. N. 121, 130–132), and heroin abuse (5–25%) (121, 133). Substance use often correlates with mood states in BD, potentially altering the clinical presentation and leading to a higher prevalence of “mixed” states (134, 135). It may also increase the risk of mood switching when patients are treated with antidepressants (136). Beyond psychiatric complications, SUD is linked to higher medical comorbidity, including HIV infection (137–139), as well as an increased risk of suicide (98, 140).
- Many BD patients experience **severe disability,** and only a minority achieve full functional recovery (71, 141–146). As early as 1990, the World Health Organization (WHO) ranked BD among the top 10 most disabling conditions in terms of disability-adjusted life years (DALYs), highlighting its devastating impact on general health, employment, relationships, education, and overall quality of life (147–151). Studies show that at least one major area of life (e.g., work, social life, or family life) is significantly impaired in 52–54% of BD patients, while in 37%, disability affects two or more areas (152). Specifically, BD-I patients are reported to be completely unable to perform work-related tasks 30% of the time, while BD-II patients experience this level of impairment 20% of the time (153). A key observation is that disability in BD is more strongly correlated with depressive symptoms—even when these symptoms are subsyndromal (5, 69, 152, 154–163). Additionally, cognitive and functional impairments persist even during euthymic periods (157, 160, 161).
- The **quality of life** (QoL) of BD patients is closely linked to current depressive symptoms, whether they meet full diagnostic criteria or are subthreshold (164–166). Other key factors that negatively impact QoL include neurocognitive impairment (164, 167), the presence of psychotic symptoms (164), and daily stressors (168). However, research suggests that some BD patients in the euthymic phase may have a quality of life comparable to that of the general population (164, 168).
- Bipolar disorder does not only affect the individual—it also places a significant **burden on caregivers and family members** (149, 169–171). This burden can be categorized into: Objective burden – tangible consequences such as financial strain, job loss, hospitalizations, and divorce (172) and subjective burden – the emotional distress and psychological toll on caregivers, including feelings of exhaustion, frustration, and anxiety (171–176). Nearly all caregivers of BD patients report at least a moderate level of burden (177–181), and it is related to patient-related factors like the chronicity of the disease and high levels of impairment (182). The severity of this burden is influenced by factors such as the chronicity of the illness, levels of patient impairment, caregiver beliefs about BD (180), personality traits, and coping styles (177). The most distressing behaviors reported by caregivers include hyperactivity, irritability, withdrawal, impulsivity, aggression, and excessive spending. BD is associated with higher rates of violence compared to other psychiatric disorders, especially during acute manic or mixed episodes (183–185). Research indicates that family members are the victims in 70% of BD-related violent episodes, and in 81% of cases, these episodes were preceded by some form of provocation (185). Additionally, depression and suicidality in BD patients significantly contribute to caregiver distress (147, 172, 180, 186). Over time, the mental health of caregivers may deteriorate due to chronic stress, potentially leading them to develop depression and an increased need for mental health services themselves (181, 187–192).
- **Increased suicidality:** Suicidal thoughts and behaviors are unfortunately common in BD, with both persistent suicidal ideation and a high rate of completed suicides being key concerns (31, 193–195). Research shows that alcohol and substance use significantly increase the risk of suicide attempts in BD patients (196). This effect appears to be more pronounced in BD-I than in BD-II, likely due to higher levels of impulsivity, hostility, aggression, and an earlier onset of the disorder in BD-I patients (197, 198).
- For BD patients and their families, the **financial strain** can be overwhelming. Studies consistently show that BD is one of the most expensive mental disorders—both in terms of insurance costs and out-of-pocket expenses for patients and their loved ones (199). When compared to other psychiatric conditions, BD ranks among the most costly, not only in mental health care but across all areas of medicine (200–206). Interestingly, BD remains the most expensive disorder in nearly every category of health benefits, yet a small group of patients (2.4%) accounts for 20% of the total costs. This cost disparity isn’t due to BD treatment alone but rather the high costs of treating medical comorbidities that frequently accompany the disorder (207, 208). This big difference in the costs is caused not because of the treatment of the primary mental diagnosis but because of comorbid somatic conditions (209).
- **Medical conditions** in BD patients are often overlooked and undertreated—especially in those who fall within the broader bipolar spectrum (210). Multiple somatic comorbidity seems to be the rule rather than the exception with BD patients suffering from an average of 2.7 or more medical conditions (211–213) and facing up to four times higher healthcare costs compared to those without mental illness, largely due to medical comorbidities (200, 209, 214, 215). Tragically, their life expectancy is reduced by approximately 30%, making it lower not only than the general population but also compared to those with other psychiatric disorders (216–218). The leading cause of early death in BD is premature cardiovascular disease, but other common medical issues include endocrine disorders, gastrointestinal problems, and chronic pain (219). Depending on the study, anywhere from 11.5% to 75.7% of BD patients suffer from at least one physical health condition, with most experiencing multiple comorbidities—on average, 2.7 or more medical conditions per patient (77, 83, 84, 88, 111, 137, 212, 220–229).
- The concept of **staging** in BD aims to define the severity, progression, physiological changes, and long-term impact of the disorder (230). So far, five major staging models have been proposed (231–237), but while some evidence supports these models, the research is still limited. Studies tend to have small sample sizes, and data inconsistencies make it difficult to establish a universal framework.

**Table 2:** List of the most important multiple clinical aspects of manic-depressive illness.

1.	Manic episodes
2.	Depressive episodes
3.	Mixed episodes
4.	Subthreshold manic symptoms
5.	Subthreshold depressive symptoms
6.	‘mixed’ states and ‘roughening’
7.	Mood lability/Cyclothymia/’Personality-like’ behaviour
8.	Predominant polarity
9.	Frequency of episodes/Rapid cycling
10.	Psychotic features
11.	Neurocognitive disorder
12.	Functional deficit and disability
13.	Drug/alcohol abuse
14.	Comorbid anxiety and other mental disorders
15.	Self-destructive behaviour and Suicidality

Currently, research suggests that BD follows a progression that includes:

- An asymptomatic “at-risk” phase – when an individual may be vulnerable to developing BD but has not yet shown clear symptoms.
- A non-specific prodromal phase – an early warning period where symptoms emerge but are not yet distinct enough to diagnose BD. This phase appears to overlap with other psychiatric disorders, making prediction difficult.
- An early stage of full-blown BD – characterized by well-defined episodes, minimal inter-episode symptoms, good response to treatment, and low disability.
- A late stage of BD – a more chronic and treatment-resistant phase, often featuring depressive predominant polarity, psychotic symptoms, and significant disability.

One major gap in research is the lack of studies on treatment effectiveness in the later stages of BD, which leaves clinicians with limited guidance on how to best manage patients with chronic, severe forms of the disorder (238). There are only a few exceptions where treatments specifically for late-stage BD have been explored (239).

## 3 Therapeutic challenges

As already said and elaborated, BD is a complex, long-term condition with an unpredictable course (240). Different aspects of the disorder respond to different medications, yet many of these treatments come with the risk of triggering the opposite mood state—for example, a medication that helps with depression may induce mania, and vice versa (241, 242). For decades, BD treatment has centered around the broad idea of “mood stabilizers”, though, in reality, this category includes only a small number of medications (243–246). More recently, research has suggested that targeting specific receptors could be a more effective approach (247). However, integrating acute-phase treatment with long-term management remains an ongoing challenge (248). One of the biggest difficulties in BD treatment is the difficulty in designing an evidence-based long-term strategy. While treatment guidelines exist, they struggle to balance current symptoms, past psychiatric history, and the potential for future relapse. This problem is comfounded by the fact that there is limited research on how to manage specific facets of BD, making it difficult for clinicians to create comprehensive, individualized treatment plans (5, 249–251). Although acute episodes (such as full-blown mania or major depression) make up only a small percentage of a patient’s lifetime, subthreshold or subclinical symptoms—mild but persistent mood disturbances—tend to dominate daily life, contributing to ongoing impairment, disability, and emotional distress (42, 252, 253). Since no single medication can fully manage all phases and symptoms of BD, most patients require a combination of treatments to achieve a reasonable quality of life (254).

Clinicians and treatment guidelines face a difficult dilemma. What should be the long-term maintenance plan for patients who initially respond well to a medication that lacks long-term safety and efficacy data—or worse, one that has negative evidence regarding its potential long-term impact on mental health? There is no clear answer, and expert opinions differ significantly. This is further complicated by the fact that most maintenance studies are conducted on “enriched samples”, meaning they focus on patients who already responded well to a specific medication during the acute phase. This creates uncertainty, especially when a first-line treatment fails or provides only partial relief. Should the clinician switch medications, potentially prolonging the patient’s suffering? Or should they add a second medication, increasing the complexity of treatment and the risk of side effects? Making these decisions requires careful consideration of various factors, including specific indications, contraindications, potential pitfalls, and patient history (244, 255–260).

The introduction of second-generation antipsychotics (SGAs) has significantly changed the landscape of BD treatment, making antipsychotics a core component of treatment guidelines. However, the role of antidepressants in BD remains controversial. While antidepressants have historically been seen as a viable treatment option for bipolar depression, particularly in Europe, recent studies question their effectiveness and even suggest that they may increase the risk of mood destabilization (261). At the same time, the value of psychosocial interventions—such as psychotherapy, lifestyle changes, and support groups—remains uncertain, as research on their effectiveness in treating specific BD symptoms is still limited (30, 262).

Residual symptoms—those that persist between major episodes—can have a major impact on a patient’s ability to function in daily life. These symptoms may interfere with access to healthcare, employment, financial stability, and even basic social support systems (263). The situation is even worse for patients with severe disability, functional decline, or poor quality of life, as they also face higher mortality rates due to medical comorbidities (227) and an increased risk of suicide (253). This not only increases the burden on caregivers and families but also drives up healthcare costs due to frequent hospitalizations and medical interventions. In many parts of the world, these challenges are worsened by discriminatory insurance policies, which limit coverage for mental health treatment and create additional financial strain on patients and their families (253, 264).

One of the biggest debates in BD treatment revolves around the very concept of “mood stabilizers.” While lithium, valproate, and carbamazepine were traditionally considered the gold standard, newer research suggests that some atypical antipsychotics—such as quetiapine and olanzapine—may meet many of the same criteria. However, no single medication is effective against all phases of BD, including manic, mixed, and depressive episodes, as well as rapid cycling. In practice, antipsychotics tend to work more quickly during acute mania and are particularly effective for psychotic symptoms. However, each medication comes with its own set of risks—for example, antipsychotics can increase the risk of metabolic syndrome, while lithium can cause kidney and thyroid issues. As already said, treating BD is inherently complex (240) and for decades, treatment was built around the concept of mood stabilizers, but in recent years, a wave of new research, particularly on atypical antipsychotics, has reshaped the field. As a result, clinicians must navigate numerous treatment considerations, including drug interactions, contraindications, and unexpected complications (244, 255–260). The definition of “mood stabilizers” remains unclear. Research does not support the idea that all traditionally labeled mood stabilizers—such as lithium, valproate, and carbamazepine—are equally effective across all phases of BD. Instead, newer studies highlight significant limitations in their effectiveness. Even more concerning, some aspects of BD may be resistant to treatment, a problem that has only recently gained attention.

Another important problem is that not only the evidence is limited concerning the treatment of specific facets and issues of BD (5, 249, 250, 265, 266), but also continued scientific training and reading are inadequate. Thus, research findings are not making it to the everyday clinical practice. Focused educational intervention might be necessary to change this attitude. Part of this problem is reflected in the common practice among clinicians to use medication based on a ‘class effect’. This means that they consider that a whole class of medications possesses a specific action. This class effect is often considered in combination with a ‘syndromal approach’ which means that irrespective of the nosological entity, a specific kind of symptoms respond to a specific class of medication.

Many clinicians also follow a “syndromal approach”, assuming that certain symptoms will respond to specific medication classes, regardless of the underlying disorder. For example, some clinicians assume that all antipsychotics work equally well for psychosis, regardless of diagnosis, and that all antidepressants are equally effective for depression. While this simplifies treatment decisions, research has repeatedly disproven this approach, particularly in BD, where the concept of mood stabilizers has been overly broad and imprecise (243). The extent to which this outdated approach still influences clinical practice worldwide is unclear, but it likely has a significant impact on treatment outcomes. If clinicians were to shift toward a more evidence-based approach, BD patients might see better long-term outcomes.

With the introduction of second-generation antipsychotics (SGAs), these medications have become a cornerstone of BD treatment, aligning with current treatment guidelines. In contrast, recent studies have cast doubt on the effectiveness of antidepressants for BD, challenging their traditional role in bipolar depression (261). Additionally, long-term treatment strategies have become more complex, as research shows that medications previously thought to be mood stabilizers may be more effective for one mood state than the other (248) Given the rapid pace of new research, it can be difficult for clinicians to stay updated and integrate these findings into their daily practice. At the same time, there is still limited data on the effectiveness of psychosocial interventions in BD. While therapy and social support play a role, their specific impact on BD symptoms remains uncertain (30, 262).

## 4 Special issues

- While it is well established that **gender-specific factors** influence the treatment and overall management of BD (267–270), research in this area remains limited. This gap is significant because the unmet needs of male and female patients may differ, potentially affecting treatment outcomes and quality of life (253, 267). Although BD-I is equally common in both males and females (253), BD-II appears to be more prevalent in females (269) as does depressive predominant polarity (45). Females with BD also tend to experience more rapid cycling, mixed episodes, and dysphoric mania (252, 253, 271–273), Additionally, they face higher rates of hypothyroidism and a greater likelihood of comorbid personality disorders (252, 253, 271–273). On the other hand, males with BD are more likely to present with suicidality, psychotic features, and a higher frequency of hospitalizations (253). One of the most significant concerns in female BD patients revolves around the reproductive cycle and its physiological impact on the disorder. Hormonal fluctuations throughout life—during menstruation, pregnancy, postpartum, and menopause—can all affect the course of BD and response to treatment. Beyond reproductive concerns, females with BD also appear to be at greater risk for specific medication-related side effects, including weight gain (274, 275) and, in severe cases, extreme obesity (276). Long-term use of certain medications, particularly those that elevate prolactin levels, may lead to a decrease in bone mineral density due to prolonged hyperprolactinemia (277). In some cases, this can even result in a hypogonadal state, further complicating overall health and treatment considerations (278). Despite these clear gender-based differences in symptoms, treatment response, and side effects, research on how to tailor BD management by gender remains insufficient. Addressing these knowledge gaps could lead to more effective, personalized treatment strategies that better meet the unique needs of both males and females.
- One of the most pressing concerns for females with BD is the risk of **unplanned pregnancy** (279). Given the potential impact of pregnancy on both the course of BD and treatment safety, females of childbearing age should receive comprehensive counseling on contraceptive options, medication interactions, and the effects of pregnancy and childbirth on their mental health. Discussions should also cover safe treatment options during pregnancy and breastfeeding, as well as the emotional and physical stress of pregnancy and parenting. Additionally, the potential risks and benefits of specific medications during different stages of pregnancy should be thoroughly explored to help patients make informed decisions (280, 281). Certain BD medications—including carbamazepine, oxcarbazepine, lamotrigine, and topiramate—are known to increase the clearance rate of oral contraceptives, potentially reducing their effectiveness. Females taking these medications may require dose adjustments or alternative contraceptive strategies as part of their standard care plan. Failing to account for these interactions can lead to contraceptive failure, increasing the risk of unintended pregnancy and associated complications. The postpartum period is one of the most vulnerable times for women with BD, with the highest risk of illness exacerbation occurring within the first 90 days after delivery (269, 282–288). This period requires careful monitoring and, in many cases, preventive treatment strategies to reduce the likelihood of severe mood episodes, which could impact both maternal well-being and infant care. Given these complexities, reproductive health should be an integral part of BD management for women, ensuring they receive personalized guidance on contraception, pregnancy planning, and postpartum care to minimize risks and promote stability.
- There is not much data concerning the **point of view of psychiatrists and therapists** in general on the unmet needs in the treatment of BD patients. However, mental health professionals generally agree that both acute episode management and long-term treatment could be improved by focusing on better treatment effectiveness, increased patient adherence, and enhanced long-term safety in maintenance therapy. Among BD patients, those with comorbid alcohol and/or substance use disorders are seen as having the greatest unmet needs, followed closely by those who experience rapid cycling (289). These populations present unique challenges, often requiring more intensive and specialized care, yet existing treatment strategies may not adequately address their specific needs. Surprisingly, only half of surveyed psychiatrists considered treatment guidelines to be an essential part of their day-to-day clinical decision-making. Even more unexpectedly, they reported that clinical trial findings had the least influence on their treatment choices. Additionally, only about one-third of clinicians were familiar with large-scale practical clinical trials or with scientific organizations and associations related to BD (290, 291). These findings highlight a critical gap between research and clinical practice, suggesting a need for greater dissemination of evidence-based knowledge and better integration of research findings into real-world treatment approaches. Bridging this gap could help ensure that patients receive the most effective, scientifically supported care while also addressing the complex challenges that come with managing BD.
- **The point of view of patients and caregivers** might vary considerably from the point of view of mental health professionals (292). One of the key factors contributing to poor treatment adherence is that clinical research often fails to focus on the unmet needs that patients themselves experience. As a result, real-world challenges are not always addressed, leaving gaps between what research prioritizes and what truly impacts daily life for those living with BD (293). There is also a significant disconnect between how mental health professionals interpret the evidence supporting different BD treatments and how patients perceive the impact of these treatments in their own lives (294). This mismatch can lead to frustration, dissatisfaction, and disengagement from care, making it even harder to achieve long-term stability. If the true measure of treatment success is based on patients’ self-reported quality of life, research presents a concerning reality. Studies show that individuals with severe mental illness, including BD, frequently report dissatisfaction with their social lives, overall health, and the level of support they receive. Many express that their unmet needs go beyond medication, extending to case management services, social and recreational opportunities, and vocational rehabilitation—all of which are crucial for achieving meaningful recovery and reintegration into society (295). Bridging the gap between clinical priorities and real-world patient needs requires a more patient-centered approach, ensuring that treatment strategies focus not only on symptom control but also on improving overall well-being, daily functioning, and quality of life.
- Poor **treatment adherence** is a major challenge in mental health care, particularly in BD, where it is strongly linked to worse outcomes (296, 297) Depending on how adherence is defined and the setting in which it is studied, research suggests that between one-third and two-thirds of BD patients do not consistently follow their prescribed treatment plans (297–299). One of the primary reasons for non-adherence is the side effects of medications, which can be difficult for patients to tolerate over long periods. Additionally, some individuals are reluctant to give up the experience of manic or hypomanic episodes, especially hypomania, which can bring increased energy, creativity, and euphoria—making it difficult for patients to fully commit to treatment that dampens these states (300). Another significant barrier to adherence is a lack of understanding about BD management. Many patients and their families do not fully grasp the long-term nature of the disorder, the importance of maintenance treatment, or the need for regular follow-up care. Without this awareness, treatment adherence can become inconsistent, increasing the risk of relapse and worsening symptoms (22). Addressing these challenges requires a combination of patient education, open communication about side effects, and individualized treatment plans that consider both clinical effectiveness and patient preferences. A more collaborative approach between patients, families, and healthcare providers may help improve adherence and lead to better long-term stability and quality of life for those living with BD.

## 5 Defining the clinical parameters to take into consideration

The key clinical and therapeutic challenges in BD have been outlined above. However, in real-world practice, these challenges often appear in unique and unpredictable combinations, which do not always fit neatly into the categories defined by modern classification systems. This makes it difficult to apply a one-size-fits-all approach to diagnosis and treatment. While it would be ideal to treat the full spectrum of symptoms as a whole rather than focusing on specific symptom clusters, current research does not always provide enough evidence to support this broader approach. Despite these limitations, it remains crucial to carefully examine the available literature when developing treatment guidelines, ensuring that they directly address these real-world complexities and provide practical solutions for clinicians.

## 6 Search of the literature and type of studies

There are three primary approaches to developing a knowledge base for BD treatment guidelines (301):

### 6.1 Expert opinion

This method is straightforward and convenient, relying on the insights of experienced professionals. However, it comes with significant risks, including the reinforcement of outdated assumptions, personal biases, and treatment approaches that may not align with the latest research findings.

### 6.2 Clinician surveys

Gathering input from a broad range of practicing clinicians can result in practical, real-world guidelines that reflect the challenges of everyday patient care. However, this approach is also prone to bias, as it may be influenced by individual experiences, unscientific beliefs, and variations in clinical training.

### 6.3 Systematic literature reviews

Examining existing research is the most scientifically rigorous way to develop guidelines, though it can take various forms. In the past, selective literature reviews were common, but today, systematic reviews are the gold standard. These reviews aim to incorporate all relevant research, minimizing personal bias and ensuring greater scientific credibility and broader acceptance. The PRISMA method is widely recognized as the most reliable approach for conducting systematic reviews and reporting of their process and results, as it ensures transparency, comprehensiveness, and methodological rigor (302–305).

While each approach has its strengths and limitations, the most reliable and widely accepted guidelines are those that rely on systematic reviews, ensuring that treatment recommendations are based on the best available evidence rather than personal or anecdotal experience. When conducting literature reviews to develop evidence-based treatment guidelines for BD, different types of research papers can be targeted. However, the most critical sources of information are Randomized Controlled Trials (RCTs). These studies may be either placebo-controlled or involve head-to-head comparisons with established treatment options. They can also focus on monotherapy (a single treatment) or combination strategies, such as add-on therapy or polypharmacy.

The distinction between add-on therapy and combination therapy is important. Combination therapy is tested in a general patient population, meaning the study includes both treatment-responsive and treatment-resistant patients. In contrast, add-on therapy specifically involves patients who have already shown resistance to treatment. A second agent is then added to determine whether it enhances effectiveness.

Another crucial aspect of interpreting research findings is understanding the difference between “failed” and “negative” trials”. A failed trial occurs when a study does not detect a positive treatment effect, even if one actually exists. This often happens due to issues such as an inappropriate study sample, such as testing a treatment on chronic patients who may not respond to the intervention. In contrast, a negative trial occurs when a treatment is genuinely ineffective, as determined by a well-designed study with an appropriate patient sample and methodology.

A classic example of a failed study is a three-arm study, where one group receives the new treatment, another receives an established treatment, and the third receives a placebo. If neither the new treatment nor the established treatment shows a meaningful difference from placebo, the trial is considered failed rather than negative—since it is likely that factors such as poor study design or patient selection interfered with the results. Unfortunately, outside of three-arm studies, it is extremely difficult—if not impossible—to distinguish between failed and negative studies in two-arm trials (where only the new treatment and placebo are compared). This often leads to the interchangeable use of the terms “failed” and “negative”, even though they describe different scenarios.

Beyond primary RCTs, *post-hoc* analyses can provide valuable insights that may not be explicitly addressed in the original study publication. However, a major limitation is that most *post-hoc* analyses are not pre-registered, making them vulnerable to selective reporting bias—where only the most favorable or significant findings are published.

Meta-analyses are another important source of information, but they are often overvalued. The sheer number of meta-analytical studies being published today is overwhelming, and many are of poor quality, sometimes leading to misleading conclusions. One common issue is that meta-analyses using raw scores instead of standardized mean differences (ratio of raw score change to standard deviation) are highly likely to produce misleading results because very often, large differences in terms of raw scores are accompanied by large standard deviations; the use of standardized mean difference might even reverse the results. Additionally, when studies vary significantly in their methodology, the combined conclusions from meta-analyses can differ both from each other and from the original findings of RCTs (306, 307).

Unpublished studies can sometimes be found in research repositories, but interpreting their results requires expertise. Many studies remain unpublished or are canceled due to negative interim findings, insufficient funding, or recruitment challenges. While these studies can still offer valuable insights, they should be approached with caution and critical analysis to determine why they were never formally published.

In summary, while systematic reviews of the literature provide essential insights for treatment guidelines, not all studies carry the same weight. RCTs remain the gold standard, but understanding nuances such as failed vs. negative trials, *post-hoc* biases, and limitations of meta-analyses is crucial for ensuring that treatment recommendations are truly evidence-based and clinically relevant.

To ensure a comprehensive and reliable review of the literature when developing BD treatment guidelines, search strategies should follow a structured approach that includes multiple sources and verification methods.

- Using Appropriate Keywords – A well-defined keyword strategy is essential to capture all relevant studies on BD treatments.
- Searching Key Research Databases – At a minimum, literature reviews should include searches in major medical and psychological research repositories, such as PubMed/Medline, Scopus, and PsycINFO. These databases contain peer-reviewed studies, systematic reviews, and meta-analyses that form the backbone of evidence-based guidelines.
- Reviewing Clinical Trial Registries – Websites that list clinical trials should also be searched, including ClinicalTrials.gov (http://clinicaltrials.gov) and Clinical Study Results (http://www.clinicalstudyresults.org). Additionally, the official websites of pharmaceutical companies producing medications for BD should be reviewed. These sources provide original pre-registered study protocols, detailing the primary and secondary outcomes of clinical trials. Such information can help identify cases of misleading reporting in published studies—for instance, the discrepancies seen in publications on lamotrigine for acute bipolar depression (308).
- Examining Reference Lists of Relevant Reviews and Guidelines – Reviewing the citations in existing systematic reviews and previously published treatment guidelines can help identify key studies that may not appear in a standard database search.
- Determining Language Inclusion Criteria – A decision must be made about whether to restrict searches to English-language publications or include studies in other languages, depending on the availability of resources for accurate translation. Important findings from non-English sources could contribute valuable insights if they can be reliably translated.
- Seeking Additional Unpublished Data – In some cases, unpublished research, particularly from pharmaceutical manufacturers or study authors, can provide critical information that is missing from published literature. These sources may contain data from studies that were never published due to negative results, funding issues, or recruitment difficulties, offering a more complete picture of treatment effectiveness and safety.

## 7 Methods to grade the findings in the literature

The process of grading medical evidence and formulating clinical recommendations has been in use since the early 1980s (309). All grading systems aim to assess the quality of available data and determine how confidently the benefits of a treatment outweigh its risks. Factors such as patient values and preferences are also considered, though in this particular framework, cost was not taken into account by the workgroup.

In 1992, a five-step approach was introduced to streamline individual-level clinical decision-making, and by 2005, it was formally published as a structured guideline (310). These five steps include:

- Formulating a Clear, Answerable Question – The first step is to define a precise and well-structured question that avoids ambiguity and uncertainty. A well-formulated question ensures that research efforts are focused and effective in addressing specific clinical concerns (311, 312).
- Conducting a Systematic Search for Evidence – A comprehensive and methodically structured search should be conducted to identify all relevant research on the topic, ensuring that no key evidence is overlooked (313).
- Critically Reviewing and Classifying the Evidence – Once relevant studies are gathered, they must be carefully evaluated for quality, considering factors such as systematic errors, different types of bias, confounding factors, reliability, and validity. Additionally, the clinical significance and generalizability of the findings must be taken into account, as results from a highly controlled study may not always translate directly to real-world clinical practice (314, 315).
- Applying the Findings in Clinical Practice – After assessing the evidence, the results must be translated into practical treatment recommendations, ensuring they align with patient needs, safety considerations, and therapeutic goals.
- Evaluating Performance and Outcomes – Finally, it is essential to monitor and assess how well the implemented guidelines perform in actual clinical practice. This includes tracking patient outcomes, treatment adherence, and any emerging concerns, allowing for continuous improvement and refinement of recommendations (316–319).

By following this structured approach, treatment guidelines can be developed in a way that ensures scientific rigor, clinical relevance, and practical applicability, ultimately improving the quality of care for individuals living with bipolar disorder.

Evaluating the quality of evidence is a crucial step in developing treatment guidelines for BD. The strength of evidence is determined by how well studies minimize biases that can distort research findings. As already discussed, the gold standard in medical research includes triple-blind, placebo-controlled trials with allocation concealment and complete follow-up in a homogeneous patient population. These studies are considered to provide the highest level of evidence, whereas case reports rank the lowest. While expert opinion can be valuable in shaping guidelines, it should not be considered a source of scientific evidence (320).

Several **grading systems** have been developed by various organizations to assess the quality of evidence. Among the most widely used are:

- The U.S. Preventive Services Task Force (USPSTF) – A system designed to evaluate the strength of clinical evidence and inform preventive healthcare recommendations (321, 322).
- The Oxford Centre for Evidence-Based Medicine (OCEBM) Levels of Evidence – A framework useful for grading diagnostic tests, prognostic markers, and treatment risks (323). This system played a role in the development of the BCLC staging system for hepatocellular carcinoma in Canada (324).
- The PORT Method (Patient Outcomes Research Team) – Used by the World Federation of Societies of Biological Psychiatry (WFSBP) in formulating their bipolar disorder guidelines (325) (326–328). In 1992, the Agency for Health Care Policy and Research (AHCPR) and the National Institute of Mental Health (NIMH) collaborated to establish the PORT for Schizophrenia, adopting similar criteria to those used in the AHCPR Depression Guidelines.

Among modern grading systems, the GRADE Method (Grading of Recommendations Assessment, Development, and Evaluation) is one of the most widely adopted approaches for guideline development (329, 330) A key feature of GRADE is that it separates the quality of evidence from the strength of recommendations. It emphasizes the importance of defining a clear clinical question, including four essential components: patient population, intervention, comparison, and outcomes of interest (331) It also categorizes outcomes based on their relevance to clinical decision-making, prioritizing those that are critical for treatment recommendations over those that are less significant (332).

The GRADE system evaluates evidence quality based on:

- Study limitations (e.g., lack of allocation concealment or blinding).
- Inconsistency of results.
- Indirectness of evidence.
- Imprecision in findings.
- Reporting bias (333–338).

Under certain conditions, evidence quality can be upgraded—for example, if a study demonstrates an exceptionally strong treatment effect (339). While GRADE provides a robust method for grading a wide range of evidence sources, it is less effective for evaluating datasets that focus solely on RCTs—such as those used in the current bipolar disorder guidelines. According to GRADE criteria, the evidence supporting the current guideline effort is considered high quality, with only two potential limitations: large losses to follow-up and early trial termination due to treatment benefits or failure to report outcomes. The GRADE method provides guidance to grade the data from a variety of sources (340), but it is not sensitive for datasets that focus solely on RCTs like the dataset of the current workgroup. According to the GRADE grading system, all the data included in the current effort to develop guidelines are of high quality. From the limitations recognized by the GRADE (lack of allocation concealment, lack of blinding, large losses to follow-up, failure to adhere to an intention to treat analysis and stopping early for benefit or failure to report outcomes) only large losses to follow-up and stopping early for benefit or failure to report outcomes could apply to the current study.

The most recent grading system was developed by the CINP Bipolar Guidelines Workgroup (341). This method was specifically designed to evaluate evidence from RCTs, *post-hoc* analyses, and meta-analyses, as no existing grading system had been developed for this purpose. While traditional grading systems rank RCTs and meta-analyses as the highest levels of evidence, they do not distinguish between conflicting results, inconsistencies between RCTs and meta-analyses, or findings derived only from secondary outcomes. The CINP system addresses these gaps by integrating a detailed evaluation of treatment efficacy across studies.

A comparative overview of these grading methods is presented in **Table 3**, while **Table 4** summarizes the different approaches for developing treatment recommendations.

**Table 3:** Comparative presentation of different grading methods.

USPSTF	OCEBM	GRADE	PORT	CINP
	Systematic review of randomized trials or n-of-1 trials	High quality	Level A: Good research-based evidence, with some expert opinion, to support the recommendation	Level1: Good research-based evidence, supported by at least 2 placebo controlled studies of sufficient magnitude and good quality. In case of the presence of negative RCTs, positive RCTs should outnumber negative ones
Level I: Evidence obtained from at least one properly designed randomized controlled trial.	Randomized trial or observational study with dramatic effect			Level 2: Fair research-based evidence, from one randomised, double-blind placebo controlled trial. Also in case one or more trials exist, however, they fail to fulfil all the criteria above (e.g., very small sample size or no placebo control) as well as in case of positive meta-analysis alone.
				Level 3: Some evidence from comparative studies without placebo arm or from *post-hoc* analyses.
Level II-1: Evidence obtained from well-designed controlled trials without randomization.		Medium quality	Level B: Fair research-based evidence, with substantial expert opinion, to support the recommendation	Level 4: Inconclusive data or poor quality of RCTs
	Non-randomized controlled cohort/follow-up study	Low quality		
Level II-2: Evidence obtained from well-designed cohort or case-control analytic studies, preferably from more than one center or research group.	Case-series, case-control studies, or historically controlled studies	Very low quality		
Level II-3: Evidence obtained from multiple time series designs with or without the intervention. Dramatic results in uncontrolled trials might also be regarded as this type of evidence.				
	Mechanism-based reasoning		Level C: Recommendation based primarily on expert opinion, with minimal research-based evidence, but significant clinical experience	
Level III: Opinions of respected authorities, based on clinical experience, descriptive studies, or reports of expert committees.				
				Level 5: negative data

USPSTF, U.S. Preventive Services Task Force.

OCEBM, Oxford (UK) Center for Evidence Based Medicine.

GRADE, Grading of Recommendations Assessment, Development and Evaluation) for the development of guidelines.

PORT, Patient Outcomes Research Team.

CINP, International College of Neuropsychopharmacology.

**Table 4:** Comparative presentation of recommendation methods.

USPSTF	GRADE	CINP
Level A: Good scientific evidence suggests that the benefits of the clinical service substantially outweigh the potential risks.	Strong	Good or fair research-based evidence (level 1 or 2) plus very good tolerability (level 1)
Level B: At least fair scientific evidence suggests that the benefits of the clinical service outweighs the potential risks.		
Level C: At least fair scientific evidence suggests that there are benefits provided by the clinical service, but the balance between benefits and risks are too close for making general recommendations.	Weak	Good or fair research-based evidence (level 1 or 2) plus moderate tolerability (level 2)
Level D: At least fair scientific evidence suggests that the risks of the clinical service outweighs potential benefits.		Some evidence from comparative studies without placebo arm or from *post-hoc* analyses (level 3) plus very good or moderate tolerability (level 1 or 2).
Level I: Scientific evidence is lacking, of poor quality, or conflicting, such that the risk versus benefit balance cannot be assessed.		Inconclusive data or poor quality of RCTs (level 4) plus poor tolerability (level 3)
		Not recommended

USPSTF, U.S. Preventive Services Task Force.

GRADE, Grading of Recommendations Assessment, Development and Evaluation) for the development of guidelines.

CINP, International College of Neuropsychopharmacology.

For example, all systems exept for the CINP are either to crude or are calibrated for use with lower quality data. Especially the USPSTF can not distinguish between scenarios with different admixture of positive and negative RCTs and meta-analyses. The greatest problem today is to rank the evidence that come from such combinations, and only the CINP method provides three levels concerning that specific area of available evidence (301, 342).

## 8 Study design and outcome

The complexity of BD presents significant challenges in treatment research, starting with the very definitions used in clinical trials. While it is relatively straightforward to define acute episodes, whether manic, hypomanic, or depressive, other terms—such as continuation and maintenance treatment—are often used interchangeably in randomized controlled trials (RCTs), leading to confusion (51, 343). By strict definition, continuation treatment lasts up to 12 months and is intended to sustain recovery from an acute episode until the point at which the episode would have naturally resolved. In contrast, maintenance treatment is designed to prevent future episodes and typically extends for several years beyond the continuation phase. However, a major challenge is that very few patients in RCTs achieve complete remission, making it difficult to clearly distinguish relapse from recurrence and continuation from maintenance treatment (344). In RCT terminology, the terms relapse and maintenance are preferred. However, the U.S. Food and Drug Administration (FDA) accepts data from patients who have been in remission for less than two months, further blurring the line between continuation and maintenance treatment (345). Even the term relapse is problematic in BD. Traditionally, a relapse is defined as the return of symptoms of the same polarity as the original episode, usually within the first few months of improvement. However, given the polymorphic nature of BD, this definition may be too restrictive, as it excludes cases where an episode of the opposite polarity emerges early in recovery. Licensing authorities tend to accept the broader definition, which includes opposite-pole relapses as part of the overall relapse rate. This variability in definitions also complicates the ability to define treatment resistance and refractoriness in BD (5, 11, 346). Another important concept in BD research is the index episode, referring to the acute episode that leads to a patient’s enrollment in a maintenance trial. Most maintenance trials follow an enriched study design, meaning that only patients who initially responded to a specific treatment during the acute phase are included in the double-blind maintenance phase. This design has important consequences. First, it biases the study sample toward patients with a specific predominant polarity (e.g., those more prone to mania or depression). Second, it favors patients who have already shown a good response to the medication being tested. As a result, findings from these trials may not apply to the general BD population, particularly patients who do not continue with the same medication in the maintenance phase or those who require a switch to another treatment (347, 348). These limitations make it difficult to translate research findings into real-world clinical practice, underscoring the need for more inclusive study designs that reflect the diverse and unpredictable course of BD.

The majority of BD treatment research focuses on measuring changes in symptom severity using standardized rating scales. While this approach provides valuable insights into short-term symptom relief, it often overlooks other critical aspects of patient well-being, such as disability, quality of life, caregiver burden, and economic impact. These factors are just as important in determining the long-term success of treatment, yet they remain understudied in clinical trials. Most experts agree that current BD treatments are more effective at reducing symptoms than at addressing functional impairment and overall long-term outcomes (296, 349–351). This gap in treatment effectiveness is particularly concerning in bipolar depression, which is notoriously difficult to treat and associated with a high risk of suicide (10, 11, 24, 251, 350, 352, 353) and profound and lasting functional impairment (354).

Beyond its impact on mental health, bipolar depression often leads to profound and long-lasting functional impairment, making it one of the most challenging phases of BD to manage (354). To improve real-world outcomes, future research should focus not only on symptom reduction but also on strategies to enhance overall functioning, reduce disability, and improve quality of life. This shift would provide a more comprehensive understanding of treatment effectiveness and help develop more patient-centered approaches to managing BD.

The vast majority of randomized controlled trials (RCTs) in BD treatment are industry-sponsored, meaning their primary goal is to obtain regulatory approval for a medication. As a result, these studies are designed to focus on specific, standardized outcome measures that align with the approval process, rather than broader aspects of patient well-being and real-world effectiveness. In acute-phase trials, the primary outcome is almost always the change in total score on a symptom severity scale, such as the Young Mania Rating Scale (YMRS), Mania Rating Scale (MRS), Montgomery-Åsberg Depression Rating Scale (MADRS), or Hamilton Depression Rating Scale (HAM-D). Other clinical measures, such as the Clinical Global Impressions (CGI) scale or the Positive and Negative Syndrome Scale (PANSS), are typically included as secondary outcomes. Additionally, response rates (percentage of patients who show significant symptom improvement) and remission rates (patients achieving minimal symptoms) are almost always secondary outcomes. For maintenance studies, the most common primary outcome is relapse into a mood episode, which helps assess how well a treatment prevents recurrence over time. However, rarely do these studies measure broader, real-world aspects of BD, such as general impairment, neurocognitive function, social and occupational quality of life, or long-term daily functioning. While the current outcome measures are useful for determining whether a drug is effective, they often fail to capture the full spectrum of what truly matters in clinical practice. Long-term success in BD treatment is not just about symptom reduction but also about improving daily life, enhancing functional recovery, and supporting overall well-being. Expanding the scope of clinical trials to include these aspects would lead to more meaningful treatment insights and better align research with the actual needs of patients and clinicians.

While including too many assessment scales in RCTs can create challenges in trial completion, it is crucial to prioritize scales that are most relevant to everyday clinical practice. Instead of relying solely on global symptom scores, trial reports should provide detailed insights into which specific BD features and specifiers respond to a given treatment. At the same time, trial feasibility and costs must be carefully balanced against the potential research benefits. A well-designed study should incorporate clinically meaningful measures without overwhelming participants or compromising trial efficiency.

Future RCTs should focus on outcomes that reflect real-world challenges, including mixed features, anxiety, psychotic symptoms, neurocognitive impairment, and disability. Currently, data on mixed features in acute bipolar depression are scarce, with most findings on mixed episodes coming from acute mania trials. At the same time, trial design should minimize the burden on both patients and researchers by avoiding unnecessary assessments, ensuring that RCTs remain both comprehensive and feasible.

One key concern in BD research is the duration of the continuation phase before transitioning into maintenance treatment, which is often too short to ensure long-term stability. This issue is particularly evident in acute-phase studies, especially for bipolar depression trials. A clear example is seen in studies on aripiprazole, where results were positive at week 6 but negative by the study endpoint at week 8 (355). This suggests that at least 8 weeks is needed in acute bipolar depression trials to capture true and lasting improvement. However, some medications have still gained approval based on studies as short as 6 weeks, raising concerns about the adequacy of current trial durations in assessing long-term effectiveness (356).

While enriched study designs help determine whether a medication remains effective long-term for patients who initially responded during the acute phase, they do not clarify whether it offers broader prophylactic benefits—particularly for patients who responded to a different treatment during the acute phase. Although many acute-phase treatments seem to provide ongoing benefits in maintenance therapy, it remains uncertain whether this applies to all medications. As a result, the generalizability of maintenance treatment efficacy beyond those who initially responded to a given agent is still largely unknown.

A three-week study duration for acute mania is likely insufficient, yet it remains the standard in most randomized controlled trials (RCTs). A more effective approach would be a 12-week study design for both acute mania and bipolar depression trials, allowing researchers to assess both manic and depressive symptoms, which frequently co-occur. While the use of placebo controls is scientifically valid, including a third arm with an active comparator would enhance assay sensitivity, providing more meaningful comparisons and improving the reliability of study findings (357).

Despite the availability of data, both authors and manufacturers often choose not to disclose certain findings. This includes key outcomes such as a treatment’s impact on core manic or depressive symptoms, mixed features, psychotic symptoms, and rapid cycling. In many cases, only p-values are reported without means and standard deviations, while in other instances, means are provided without statistical significance markers, leading to confusion in interpretation. Additionally, some studies report total scale scores, such as the PANSS total score, without offering a detailed breakdown of symptom domains, making it difficult to assess specific treatment effects. Another concern is the lack of transparency in sample sizes. Often, data are missing for portions of the study population, leading to varying sample sizes for different outcomes—yet this is not always clearly stated. A particularly unacceptable practice is seen in studies on mixed episodes, where only the effect of treatment on manic symptoms is reported, while the impact on depressive symptoms is omitted. This selective reporting limits clinicians’ ability to make informed decisions, emphasizing the need for greater transparency and comprehensive data presentation in BD research.

Making raw data accessible to the scientific community could lead to major advancements in our understanding of BD without requiring new and costly research. A more exhaustive analysis of existing data could provide valuable insights, improve treatment strategies, and enhance the real-world applicability of findings. Additionally, open access to data would help eliminate publication bias and improve the reliability of research conclusions.

A review of the literature suggests that study results are reported inconsistently, often lacking a uniform structure despite the existence of general reporting templates (9, 301, 341, 342). This inconsistency creates significant challenges when attempting to extract data for meta-analyses. Frequently, important details are missing, such as scores on the positive symptom subscale of the PANSS, while less critical information, like the total PANSS score, is provided instead. Most studies rely on the Last Observation Carried Forward (LOCF) approach, while a smaller number use the Mixed-Effect Model Repeated Measure (MMRM) method. In some cases, results are selectively reported from either model, despite each having its own strengths and limitations (358). Another notable issue is the inconsistency in reported sample sizes across different publications of the same original study. This lack of clarity further complicates data interpretation and comparison. To improve the quality and transparency of research, it is essential for study reports to adhere to CONSORT guidelines, ensuring that data is accurately and consistently presented for both researchers and clinicians.

## 9 Development of the actual guideline

One of the biggest challenges in developing treatment guidelines for BD is that research tends to treat BD as a collection of separate, independent phases, rather than as a single, interconnected disorder. This creates a critical dilemma for both clinicians and guideline developers: how should maintenance treatment be determined if a patient responded well to an acute-phase treatment, but there is little to no data on its long-term preventive effects—or worse if existing data suggest negative outcomes in the long run? For example, consider a patient who successfully responded to haloperidol for an acute manic episode. However, if this patient’s history shows that most of their past mood episodes were depressive, depression will likely remain the predominant issue in the future. This puts the clinician in a difficult position: should they add another medication with proven efficacy in preventing depressive episodes, such as quetiapine, resulting in combination therapy? Or should they switch to monotherapy with a drug that offers prophylactic protection against both manic and depressive episodes? There is no clear answer, and expert opinions vary, especially since most maintenance trials use enriched study samples—meaning that they only include patients who initially responded to the tested medication during the acute phase. This makes it even harder to determine what to do for patients who did not respond well to first-line treatment. Should the clinician switch medications, which might prolong suffering due to delayed stabilization? Or should they add another agent, increasing the risk of polypharmacy and side effects? Future research should prioritize finding solutions to these challenges. Ideally, all treatment options should be tested across all phases and clinical features of BD, so that those with the broadest efficacy are given priority in clinical use. Of course, even when broader efficacy is established, safety and tolerability concerns can further complicate treatment decisions, making it crucial to weigh both effectiveness and long-term patient well-being (5, 11, 346).

Designing the format of treatment guidelines presents its own set of challenges. One possible approach is to develop a precise, step-by-step algorithm based entirely on scientific evidence. This algorithm would be the final stage of guideline development, following the grading of available data and treatment recommendations. Such an algorithm would be strictly data-driven, providing clear and precise treatment pathways. However, it would likely be limited in its real-world clinical applicability. There would be no flexibility to accommodate individual patient nuances, as evidence-based decision-making would take absolute priority over clinical intuition or practical considerations. While it would reflect the most current state of scientific knowledge, it would lack the adaptability needed for everyday clinical practice, making its implementation challenging. Clinicians interested in using such a model would need to understand both its strengths and its limitations. Given the complexity of bipolar disorder treatment, such an algorithm may end up being so intricate that it could only be effectively applied through a digital tool, such as a mobile application, to guide decision-making in real-time.

An alternative approach to developing BD treatment guidelines is to incorporate clinical wisdom alongside research evidence. While this method introduces the risk of biases and, in some cases, may even lead to overlooking certain research findings, it would likely be more practical, easier to adopt, and more intuitive for clinicians in real-world practice. For guidelines to be effective and widely accepted, they should be rooted in solid research evidence while also being adaptable to everyday clinical challenges. A rigid, purely data-driven model may be scientifically sound but impractical, whereas an approach that blends research with real-world insights could enhance clinical decision-making and increase usability. Although the core framework of such guidelines should remain evidence-based, their interpretation and application should avoid excessively rigid interpretations of research findings. Instead, they should be structured in a way that acknowledges the complexity of BD and allows clinicians to make well-informed, patient-centered decisions without being constrained by an overly narrow or impractical set of recommendations.

## 10 Economic considerations

Estimating the true economic cost of BD is incredibly challenging due to its highly variable and unpredictable nature. The financial burden extends far beyond the direct costs of hospitalizations and medication—it also includes the expense of healthcare infrastructure, the impact of comorbid medical conditions, and indirect costs such as out-of-pocket expenses, lost productivity due to work absences, and even premature death (359). Because BD affects multiple aspects of a person’s life, its financial toll is not easily captured by traditional healthcare cost analyses. A comprehensive assessment must account for both short-term medical expenses and long-term socioeconomic consequences, ensuring that the full burden of the disorder is properly recognized and addressed.

In the UK, the total cost of BD was estimated at £2.055 billion in 1999/2000 prices (202). Notably, the majority of this cost (86%) was due to productivity loss and unemployment, while only 10% was directly related to National Health Service (NHS) expenses. Medication costs in primary care were relatively low at £8.5 million, making up just 0.4% of the total cost and 4.3% of NHS-related costs. However, a more recent analysis found that NHS costs had doubled, with medication expenses rising disproportionately to £25.2 million, accounting for 7.4% of NHS costs (360). In the United States, medication costs were minimal throughout the 1990s but increased significantly after 2000, eventually reaching 2% of the total cost, although exact figures remain unclear (204, 361–363). In Germany, the total annual cost of BD was estimated at 5.8 billion euros, with a staggering 98% attributed to productivity loss (364). Similar estimates have been reported worldwide, though figures vary depending on prevalence rates, healthcare systems, and societal structures (365–367). These findings highlight that the economic impact of BD extends far beyond direct medical costs, with lost productivity and unemployment being the largest financial burden, reinforcing the need for effective long-term management strategies to reduce both individual and societal costs.

While medication costs make up only a small fraction of the total cost of BD (368), they play a critical role in managing the illness. Effective pharmacological treatment is the foundation of BD management, enabling the resolution of acute episodes, reducing long-term impairment, and enhancing patient insight and treatment adherence. By stabilizing symptoms, medication also allows for other therapeutic interventions, such as psychotherapy, rehabilitation, and social support, to be more effective. However, in some parts of the world and during certain periods, medication costs have risen disproportionately, raising economic concerns. While cost-containment strategies—such as prioritizing cheaper medications over newer treatments—may seem appealing, they must be approached with caution. A short-term reduction in medication expenses that disregards clinical evidence could ultimately lead to a disproportionate increase in the total cost of BD, as poorly managed treatment could result in higher rates of hospitalization, disability, and lost productivity. Balancing cost efficiency with clinical effectiveness is essential to ensuring both financial sustainability and optimal patient outcomes.

## 11 Discussion

Just as BD is a complex and demanding condition to treat, developing treatment guidelines for BD presents an equally challenging task. Compared to more linear disorders such as schizophrenia or unipolar depression, BD is inherently more variable, requiring a more nuanced approach across all aspects of research, clinical practice, and treatment planning. Its episodic nature, diverse symptomatology, and fluctuating treatment needs make it difficult to establish one-size-fits-all recommendations. As a result, BD remains one of the most challenging psychiatric conditions to address, both in clinical care and in the development of structured treatment guidelines.

Guidelines should carefully address the unmet clinical needs that exist across all phases of BD, as these represent a key priority. Treatment guidelines are only truly valuable when they lead to improved outcomes, and this improvement must come from directly tackling the gaps in current care. A review of the literature suggests that early and accurate diagnosis, along with better education for patients and their families, may be among the most pressing unmet needs in bipolar disorder. However, research has also highlighted other significant issues, not only in terms of available treatment knowledge but also in the methods used to conduct clinical research. Addressing these gaps should be a fundamental goal in the development of more effective and applicable treatment guidelines.

One key takeaway message is that existing research may already hold answers to many clinical questions, including how to tailor treatment for specific patient subgroups. This could encourage guideline developers to rely heavily—if not entirely—on hard scientific data. However, the literature often lacks exhaustive analyses, and raw data are rarely made available. Maximizing the use of already collected data could have a more immediate impact on clinical practice than conducting new studies. Given the urgency of improving treatment outcomes, making these data accessible and conducting thorough analyses should be a priority for public health.

It is becoming increasingly clear that future RCTs should follow a standardized design that captures the full complexity of bipolar disorder. This means assessing manic, depressive, and psychotic symptoms simultaneously across all phases of the illness. Standardization would help reduce biases and inconsistencies that often arise due to the way studies are currently conducted. Equally important is the need for a uniform approach to reporting results. At present, only a limited and often fragmented portion of trial findings is made available, and it is not uncommon for different reports of the same study to present slightly varying figures. This raises concerns about the overall reliability of scientific reporting and highlights the need for greater transparency. Beyond summarizing and evaluating the evidence, treatment guidelines should also serve as an educational tool, promoting best practices and ensuring that clinicians have access to clear, consistent, and reliable information to guide patient care.

How clinicians would best use guidelines is an open question and difficult to answer. Simple logic and common sense dictate that studying them and including them in their library of knowledge will, by definition, improve clinical practice since it will improve the base of knowledge one relies on, even if no specific step is followed explicitly. Additionally, trying to chart individual cases on the landscape of treatment strategies and trajectories that guidelines provide, is expected to improve, at least partially, the outcome.

In conclusion, creating treatment guidelines for bipolar disorder is a complex and demanding task. It requires a thorough review of existing literature, including uncovering and analyzing unpublished data to fully understand its implications. Beyond gathering information, the real challenge lies in weaving together the fragmented research, which often focuses on isolated aspects of the disorder, into a cohesive framework for decision-making. A well-developed guideline must bridge past knowledge with present clinical decisions while anticipating future challenges. It should not only reflect the best available evidence but also provide a practical, forward-thinking approach to managing the disorder in real-world settings.

Apart from how guidelines should handle this fragmentation, it is necessary for future research to adopt a different approach and model of trial design; this should be more longitudinal with multiple clinically informed primary outcomes and interventions (341).

## References

[1] Bauer M Glenn T Alda M Andreassen OA Angelopoulos E Ardau R . Influence of light exposure during early life on the age of onset of bipolar disorder. J Psychiatr Res. (2015) 64:1–8. doi: 10.1016/j.jpsychires.2015.03.013 25862378

[2] Bauer M Glenn T Alda M Andreassen OA Angelopoulos E Ardau R . Relationship between sunlight and the age of onset of bipolar disorder: an international multisite study. J Affect Disord. (2014) 167:104–11. doi: 10.1016/j.jad.2014.05.032 24953482

[3] Carvalho AF Hyphantis TN Taunay TC Macêdo DS Floros GD Ottoni GL . The relationship between affective temperaments, defensive styles and depressive symptoms in a large sample. J Affect Disord. (2013) 146:58–65. doi: 10.1016/j.jad.2012.08.038 22963895

[4] Fountoulakis K . Treatment guidelines. In: Fountoulakis K , editor. Bipolar Disorder: An Evidence-Based Guide to Manic Depression. Springer-Verlag Berlin Heidelberg (2015). p. 643–58.

[5] Fountoulakis KN . An update of evidence-based treatment of bipolar depression: where do we stand? Curr Opin Psychiatry. (2010) 23:19–24. doi: 10.1097/YCO.0b013e328333e132 19901836

[6] Fountoulakis KN Moeller HJ Kasper S . Personalised and precision psychiatry: what do the CINP bipolar guidelines suggest? Int J Psychiatry Clin Pract. (2019) 23:80–1. doi: 10.1080/13651501.2018.1470246 29764259

[7] Goodwin GM Haddad PM Ferrier IN Aronson JK Barnes T Cipriani A . Evidence-based guidelines for treating bipolar disorder: Revised third edition recommendations from the British Association for Psychopharmacology. J Psychopharmacol. (2016) 30:495–553. doi: 10.1177/0269881116636545 26979387 PMC4922419

[8] Yatham LN Kennedy SH Parikh SV Schaffer A Bond DJ Frey BN . Canadian Network for Mood and Anxiety Treatments (CANMAT) and International Society for Bipolar Disorders (ISBD) 2018 guidelines for the management of patients with bipolar disorder. Bipolar Disord. (2018) 20:97–170. doi: 10.1111/bdi.12609 29536616 PMC5947163

[9] Fountoulakis KN Grunze H Vieta E Young A Yatham L Blier P . The international college of neuro-psychopharmacology (CINP) treatment guidelines for bipolar disorder in adults (CINP-BD-2017), part 3: the clinical guidelines. Int J Neuropsychopharmacol. (2017) 20:180–95. doi: 10.1093/ijnp/pyw109 PMC5408976 27941079

[10] Fountoulakis KN Magiria S Siamouli M Panagiotidis P Nimatoudis I Iacovides A . A seven- year follow-up of an extremely refractory bipolar I patient. CNS Spectr. (2007) 12:733–4. doi: 10.1017/s109285290001539x 18018326

[11] Fountoulakis KN Yatham LN Grunze H Vieta E Young AH Blier P . The CINP guidelines on the definition and evidence-based interventions for treatment-resistant bipolar disorder. Int J Neuropsychopharmacol. (2020) 23:230–56. doi: 10.1093/ijnp/pyz064 PMC7177170 31802122

[12] Woo YS Bahk WM Lee JG Jeong JH Kim MD Sohn I . Korean medication algorithm project for bipolar disorder 2018 (KMAP-BP 2018): fourth revision. Clin Psychopharmacol Neurosci. (2018) 16:434–48. doi: 10.9758/cpn.2018.16.4.434 PMC6245301 30466216

[13] NICE . Bipolar disorder guidelines 2020 amendment (CG185) (2020). Available online at: https://www.nice.org.uk/Guidance/Conditions-and-diseases/Mental-health-and-behavioural-conditions/Bipolar-disorder (Accessed March 30, 2025).

[14] Malhi GS Outhred T Morris G Boyce PM Bryant R Fitzgerald PB . Royal Australian and New Zealand College of Psychiatrists clinical practice guidelines for mood disorders: bipolar disorder summary. Med J Aust. (2018) 208:219–25. doi: 10.5694/mja17.00658 29540132

[15] Grunze H Vieta E Goodwin GM Bowden C Licht RW Azorin JM . The World Federation of Societies of Biological Psychiatry (WFSBP) Guidelines for the Biological Treatment of Bipolar Disorders: Acute and long-term treatment of mixed states in bipolar disorder. World J Biol Psychiatry. (2018) 19:2–58. doi: 10.1080/15622975.2017.1384850 29098925

[16] Grunze H Vieta E Goodwin GM Bowden C Licht RW Moller HJ . The World Federation of Societies of Biological Psychiatry (WFSBP) guidelines for the biological treatment of bipolar disorders: update 2009 on the treatment of acute mania. World J Biol Psychiatry. (2009) 10:85–116. doi: 10.1080/15622970902823202 19347775

[17] Grunze H Vieta E Goodwin GM Bowden C Licht RW Moller HJ . The World Federation of Societies of Biological Psychiatry (WFSBP) Guidelines for the Biological Treatment of Bipolar Disorders: Update 2010 on the treatment of acute bipolar depression. World J Biol Psychiatry. (2010) 11:81–109. doi: 10.3109/15622970903555881 20148751

[18] Grunze H Vieta E Goodwin GM Bowden C Licht RW Moller HJ . The World Federation of Societies of Biological Psychiatry (WFSBP) guidelines for the biological treatment of bipolar disorders: update 2012 on the long-term treatment of bipolar disorder. World J Biol Psychiatry. (2013) 14:154–219. doi: 10.3109/15622975.2013.770551 23480132

[19] Fountoulakis KN . Treatment guidelines for mental disorders: reality or illusion? Psychiatriki. (2015) 26:89–92. 26197098

[20] Hirschfeld RM Lewis L Vornik LA . Perceptions and impact of bipolar disorder: how far have we really come? Results of the national depressive and manic-depressive association 2000 survey of individuals with bipolar disorder. J Clin Psychiatry. (2003) 64:161–74. doi: 10.4088/JCP.v64n0209 12633125

[21] Lewis L . The national depressive and manic-depressive association: an introduction. Biol Psychiatry. (2000) 47:692. doi: 10.1016/s0006-3223(00)00827-1 10773174

[22] Lish JD Dime-Meenan S Whybrow PC Price RA Hirschfeld RM . The National Depressive and Manic-depressive Association (DMDA) survey of bipolar members. J Affect Disord. (1994) 31:281–94. doi: 10.1016/0165-0327(94)90104-x 7989643

[23] Morselli PL Elgie R Europe G . GAMIAN-Europe/BEAM survey I–global analysis of a patient questionnaire circulated to 3450 members of 12 European advocacy groups operating in the field of mood disorders. Bipolar Disord. (2003) 5:265–78. doi: 10.1034/j.1399-5618.2003.00037.x 12895204

[24] Frye MA Gitlin MJ Altshuler LL . Unmet needs in bipolar depression. Depress Anxiety. (2004) 19:199–208. doi: 10.1002/da.20013 15274168

[25] Ketter TA . Nosology, diagnostic challenges, and unmet needs in managing bipolar disorder. J Clin Psychiatry. (2010) 71:e27. doi: 10.4088/JCP.8125tx12c 21062613

[26] Vieta E . Antidepressants in bipolar I disorder: never as monotherapy. Am J Psychiatry. (2014) 171:1023–6. doi: 10.1176/appi.ajp.2014.14070826 25272338

[27] Angst J . The bipolar spectrum. Br J Psychiatry. (2007) 190:189–91. doi: 10.1192/bjp.bp.106.030957 17329735

[28] Angst J Sellaro R Stassen HH Gamma A . Diagnostic conversion from depression to bipolar disorders: results of a long-term prospective study of hospital admissions. J Affect Disord. (2005) 84:149–57. doi: 10.1016/S0165-0327(03)00195-2 15708412

[29] Akiskal H . Mood Disorders. In: Sadock B Sadock V , editors. Comprehensive Textbook of Psychiatry, vol. I . Lippincott Williams & Wilkins, Philadelphia (2000). p. 1338–77.

[30] Fountoulakis KN Gonda X Siamouli M Rihmer Z . Psychotherapeutic intervention and suicide risk reduction in bipolar disorder: a review of the evidence. J Affect Disord. (2009) 113:21–9. doi: 10.1016/j.jad.2008.06.014 18676024

[31] Fountoulakis KN Iacovides A Fotiou F Nimatoudis J Bascialla F Ioannidou C . Neurobiological and psychological correlates of suicidal attempts and thoughts of death in patients with major depression. Neuropsychobiology. (2004) 49:42–52. doi: 10.1159/000075338 14730200

[32] Fountoulakis KN Karakatsoulis GN Abraham S Adorjan K Ahmed HU Alarcon RD . Somatic multicomorbidity and disability in patients with psychiatric disorders in comparison to the general population: a quasi-epidemiological investigation in 54,826 subjects from 40 countries (COMET-G study). CNS Spectr. (2024) 29:126–49. doi: 10.1017/S1092852924000026 38269574

[33] Gonda X Fountoulakis KN Harro J Pompili M Akiskal HS Bagdy G . The possible contributory role of the S allele of 5-HTTLPR in the emergence of suicidality. J Psychopharmacol. (2011) 25:857–66. doi: 10.1177/0269881110376693 20837566

[34] Rihmer Z Gonda X Rihmer A Fountoulakis KN . Suicidal and violent behaviour in mood disorders: A major public health problem. A review for the clinician. Int J Psychiatry Clin Pract. (2010) 14:88–94. doi: 10.3109/13651501003624712 24922467

[35] Angst J . The course of affective disorders. II. Typology of bipolar manic-depressive illness. Arch Psychiatr Nervenkr (1970). (1978) 226:65–73. doi: 10.1007/BF00344125 708228

[36] Baldessarini RJ Undurraga J Vazquez GH Tondo L Salvatore P Ha K . Predominant recurrence polarity among 928 adult international bipolar I disorder patients. Acta Psychiatr Scand. (2012) 125:293–302. doi: 10.1111/j.1600-0447.2011.01818.x 22188017

[37] Carvalho AF McIntyre RS Dimelis D Gonda X Berk M Nunes-Neto PR . Predominant polarity as a course specifier for bipolar disorder: a systematic review. J Affect Disord. (2014) 163:56–64. doi: 10.1016/j.jad.2014.03.035 24836088

[38] Carvalho AF Quevedo J McIntyre RS Soeiro-de-Souza MG Fountoulakis KN Berk M . Treatment implications of predominant polarity and the polarity index: a comprehensive review. Int J Neuropsychopharmacol. (2014) 18. doi: 10.1093/ijnp/pyu079 PMC4368897 25522415

[39] Carvalho AF Quevedo J McIntyre RS Soeiro-de-Souza MG Fountoulakis KN Berk M . Treatment implications of predominant polarity and the polarity index: a comprehensive review. Int J Neuropsychopharmacol. (2015) 18:pyu079. doi: 10.1093/ijnp/pyu079 PMC4368897 25522415

[40] Colom F Vieta E Daban C Pacchiarotti I Sanchez-Moreno J . Clinical and therapeutic implications of predominant polarity in bipolar disorder. J Affect Disord. (2006) 93:13–7. doi: 10.1016/j.jad.2006.01.032 16650901

[41] García-López A De Dios-Perrino C Ezquiaga E . P.3.e.007 Polarity of the first episode and predominant polarity in a cohort of bipolar outpatients. Eur Neuropsychopharmacol. (2009) 19:S571–1. doi: 10.1016/s0924-977x(09)70912-5

[42] Judd LL Akiskal HS Schettler PJ Coryell W Endicott J Maser JD . A prospective investigation of the natural history of the long-term weekly symptomatic status of bipolar II disorder. Arch Gen Psychiatry. (2003) 60:261–9. doi: 10.1001/archpsyc.60.3.261 12622659

[43] Leonhard K . Die prapsychotische Temperamente bei den monopolaren und bipolaren phasischen Psychosen. Psychiat Neurol (Basel). (1963) 146:105–15. 14046917

[44] Mazzarini L Pacchiarotti I Colom F Sani G Kotzalidis GD Rosa AR . Predominant polarity and temperament in bipolar and unipolar affective disorders. J Affect Disord. (2009) 119:28–33. doi: 10.1016/j.jad.2009.03.016 19346002

[45] Nivoli AM Pacchiarotti I Rosa AR Popovic D Murru A Valenti M . Gender differences in a cohort study of 604 bipolar patients: the role of predominant polarity. J Affect Disord. (2011) 133:443–9. doi: 10.1016/j.jad.2011.04.055 21620480

[46] Pacchiarotti I Nivoli AM Mazzarini L Kotzalidis GD Sani G Koukopoulos A . The symptom structure of bipolar acute episodes: in search for the mixing link. J Affect Disord. (2013) 149:56–66. doi: 10.1016/j.jad.2013.01.003 23394711

[47] Perris C d’Elia G . A study of bipolar (manic-depressive) and unipolar recurrent depressive psychoses. IX. therapy and prognosis. Acta Psychiatr Scand Suppl. (1966) 194:153–71. doi: 10.1111/j.1600-0447.1966.tb11018.x 5229226

[48] Perris C d’Elia G . A study of bipolar (manic-depressive) and unipolar recurrent depressive psychoses. X. Mortality, suicide and life-cycles. Acta Psychiatr Scand Suppl. (1966) 194:172–89. doi: 10.1111/j.1600-0447.1966.tb11019.x 5229228

[49] Quitkin FM Rabkin JG Prien RF . Bipolar disorder: are there manic-prone and depressive-prone forms? J Clin Psychopharmacol. (1986) 6:167–72. 3711367

[50] Rosa AR Andreazza AC Kunz M Gomes F Santin A Sanchez-Moreno J . Predominant polarity in bipolar disorder: diagnostic implications. J Affect Disord. (2008) 107:45–51. doi: 10.1016/j.jad.2007.07.021 17804081

[51] Tohen M Frank E Bowden CL Colom F Ghaemi SN Yatham LN . The International Society for Bipolar Disorders (ISBD) Task Force report on the nomenclature of course and outcome in bipolar disorders. Bipolar Disord. (2009) 11:453–73. doi: 10.1111/j.1399-5618.2009.00726.x 19624385

[52] Vieta E Berk M Wang W Colom F Tohen M Baldessarini RJ . Predominant previous polarity as an outcome predictor in a controlled treatment trial for depression in bipolar I disorder patients. J Affect Disord. (2009) 119:22–7. doi: 10.1016/j.jad.2009.02.028 19324419

[53] Azorin JM Kaladjian A Adida M Hantouche EG Hameg A Lancrenon S . Factors associated with rapid cycling in bipolar I manic patients: findings from a French national study. CNS Spectr. (2008) 13:780–7. doi: 10.1017/s1092852900013900 18849897

[54] Coryell W Endicott J Keller M . Rapidly cycling affective disorder. Demographics, diagnosis, family history, and course. Arch Gen Psychiatry. (1992) 49:126–31. doi: 10.1001/archpsyc.1992.01820020046006 1550465

[55] Coryell W Solomon D Turvey C Keller M Leon AC Endicott J . The long-term course of rapid-cycling bipolar disorder. Arch Gen Psychiatry. (2003) 60:914–20. doi: 10.1001/archpsyc.60.9.914 12963673

[56] Cruz N Vieta E Comes M Haro JM Reed C Bertsch J . Rapid-cycling bipolar I disorder: course and treatment outcome of a large sample across Europe. J Psychiatr Res. (2008) 42:1068–75. doi: 10.1016/j.jpsychires.2007.12.004 18262204

[57] Dittmann S Biedermann NC Grunze H Hummel B Scharer LO Kleindienst N . The Stanley Foundation Bipolar Network: results of the naturalistic follow-up study after 2.5 years of follow-up in the German centres. Neuropsychobiology. (2002) 46 Suppl 1:2–9. doi: 10.1159/000068018 12571425

[58] Dunner DL Fieve RR . Clinical factors in lithium carbonate prophylaxis failure. Arch Gen Psychiatry. (1974) 30:229–33. doi: 10.1001/archpsyc.1974.01760080077013 4589148

[59] Garcia-Amador M Colom F Valenti M Horga G Vieta E . Suicide risk in rapid cycling bipolar patients. J Affect Disord. (2009) 117:74–8. doi: 10.1016/j.jad.2008.12.005 19121546

[60] Hajek T Hahn M Slaney C Garnham J Green J Ruzickova M . Rapid cycling bipolar disorders in primary and tertiary care treated patients. Bipolar Disord. (2008) 10:495–502. doi: 10.1111/j.1399-5618.2008.00587.x 18452445 PMC3544929

[61] Kramlinger KG Post RM . Ultra-rapid and ultradian cycling in bipolar affective illness. Br J Psychiatry. (1996) 168:314–23. doi: 10.1192/bjp.168.3.314 8833685

[62] Kukopulos A Reginaldi D Laddomada P Floris G Serra G Tondo L . Course of the manic-depressive cycle and changes caused by treatment. Pharmakopsychiatr Neuropsychopharmakol. (1980) 13:156–67. doi: 10.1055/s-2007-1019628 6108577

[63] Lee S Tsang A Kessler RC Jin R Sampson N Andrade L . Rapid-cycling bipolar disorder: cross-national community study. Br J Psychiatry. (2010) 196:217–25. doi: 10.1192/bjp.bp.109.067843 PMC2830056 20194545

[64] Nurnberger J Jr. Guroff JJ Hamovit J Berrettini W Gershon E . A family study of rapid-cycling bipolar illness. J Affect Disord. (1988) 15:87–91. doi: 10.1016/0165-0327(88)90013-4 2970497

[65] Schneck CD Miklowitz DJ Calabrese JR Allen MH Thomas MR Wisniewski SR . Phenomenology of rapid-cycling bipolar disorder: data from the first 500 participants in the Systematic Treatment Enhancement Program. Am J Psychiatry. (2004) 161:1902–8. doi: 10.1176/ajp.161.10.1902 15465989

[66] Schneck CD Miklowitz DJ Miyahara S Araga M Wisniewski S Gyulai L . The prospective course of rapid-cycling bipolar disorder: findings from the STEP-BD. Am J Psychiatry. (2008) 165:370–377; quiz 410. doi: 10.1176/appi.ajp.2007.05081484 18198271

[67] Yildiz A Sachs GS . Characteristics of rapid cycling bipolar-I patients in a bipolar speciality clinic. J Affect Disord. (2004) 79:247–51. doi: 10.1016/S0165-0327(02)00350-6 15023502

[68] Burdick KE Goldberg JF Harrow M . Neurocognitive dysfunction and psychosocial outcome in patients with bipolar I disorder at 15-year follow-up. Acta Psychiatr Scand. (2010) 122:499–506. doi: 10.1111/j.1600-0447.2010.01590.x 20637012 PMC2980854

[69] Mur M Portella MJ Martinez-Aran A Pifarre J Vieta E . Influence of clinical and neuropsychological variables on the psychosocial and occupational outcome of remitted bipolar patients. Psychopathology. (2009) 42:148–56. doi: 10.1159/000207456 19276630

[70] Martinez-Aran A Vieta E Colom F Reinares M Benabarre A Torrent C . Neuropsychological performance in depressed and euthymic bipolar patients. Neuropsychobiology. (2002) 46 Suppl 1:16–21. doi: 10.1159/000068016 12571428

[71] Martinez-Aran A Vieta E Torrent C Sanchez-Moreno J Goikolea JM Salamero M . Functional outcome in bipolar disorder: the role of clinical and cognitive factors. Bipolar Disord. (2007) 9:103–13. doi: 10.1111/j.1399-5618.2007.00327.x 17391354

[72] Cullen B Ward J Graham NA Deary IJ Pell JP Smith DJ . Prevalence and correlates of cognitive impairment in euthymic adults with bipolar disorder: A systematic review. J Affect Disord. (2016) 205:165–81. doi: 10.1016/j.jad.2016.06.063 27449549

[73] Tsitsipa E Fountoulakis KN . The neurocognitive functioning in bipolar disorder: a systematic review of data. Ann Gen Psychiatry. (2015) 14:42. doi: 10.1186/s12991-015-0081-z 26628905 PMC4666163

[74] Garriga M Pacchiarotti I Kasper S Zeller SL Allen MH Vazquez G . Assessment and management of agitation in psychiatry: Expert consensus. World J Biol Psychiatry. (2016) 17:86–128. doi: 10.3109/15622975.2015.1132007 26912127

[75] Maj M . Psychiatric comorbidity”: an artefact of current diagnostic systems? Br J Psychiatry. (2005) 186:182–4. doi: 10.1192/bjp.186.3.182 15738496

[76] Dell’Osso B Buoli M Bortolussi S Camuri G Vecchi V Altamura AC . Patterns of Axis I comorbidity in relation to age in patients with Bipolar Disorder: a cross-sectional analysis. J Affect Disord. (2011) 130:318–22. doi: 10.1016/j.jad.2010.10.008 21074273

[77] Krishnan KR . Psychiatric and medical comorbidities of bipolar disorder. Psychosom Med. (2005) 67:1–8. doi: 10.1097/01.psy.0000151489.36347.18 15673617

[78] Leverich GS Altshuler LL Frye MA Suppes T Keck PE Jr. McElroy SL . Factors associated with suicide attempts in 648 patients with bipolar disorder in the Stanley Foundation Bipolar Network. J Clin Psychiatry. (2003) 64:506–15. doi: 10.4088/jcp.v64n0503 12755652

[79] Mantere O Melartin TK Suominen K Rytsala HJ Valtonen HM Arvilommi P . Differences in Axis I and II comorbidity between bipolar I and II disorders and major depressive disorder. J Clin Psychiatry. (2006) 67:584–93. doi: 10.4088/jcp.v67n0409 16669723

[80] McElroy SL Altshuler LL Suppes T Keck PE Jr. Frye MA Denicoff KD . Axis I psychiatric comorbidity and its relationship to historical illness variables in 288 patients with bipolar disorder. Am J Psychiatry. (2001) 158:420–6. doi: 10.1176/appi.ajp.158.3.420 11229983

[81] Preti A Vrublevska J Veroniki AA Huedo-Medina TB Fountoulakis KN . Prevalence, impact and treatment of generalised anxiety disorder in bipolar disorder: a systematic review and meta-analysis. Evid Based Ment Health. (2016) 19:73–81. doi: 10.1136/eb-2016-102412 27405742 PMC10699460

[82] Sasson Y Chopra M Harrari E Amitai K Zohar J . Bipolar comorbidity: from diagnostic dilemmas to therapeutic challenge. Int J Neuropsychopharmacol. (2003) 6:139–44. doi: 10.1017/S1461145703003432 12890307

[83] Strakowski SM Tohen M Stoll AL Faedda GL Goodwin DC . Comorbidity in mania at first hospitalization. Am J Psychiatry. (1992) 149:554–6. doi: 10.1176/ajp.149.4.554 1348163

[84] Subramaniam M Abdin E Vaingankar JA Chong SA . Prevalence, correlates, comorbidity and severity of bipolar disorder: results from the Singapore Mental Health Study. J Affect Disord. (2013) 146:189–96. doi: 10.1016/j.jad.2012.09.002 23017543

[85] Tohen M Zarate CA Jr. Hennen J Khalsa HM Strakowski SM Gebre-Medhin P . The McLean-Harvard First-Episode Mania Study: prediction of recovery and first recurrence. Am J Psychiatry. (2003) 160:2099–107. doi: 10.1176/appi.ajp.160.12.2099 14638578

[86] Vieta E Colom F Corbella B Martinez-Aran A Reinares M Benabarre A . Clinical correlates of psychiatric comorbidity in bipolar I patients. Bipolar Disord. (2001) 3:253–8. doi: 10.1034/j.1399-5618.2001.30504.x 11903208

[87] Bellani M Hatch JP Nicoletti MA Ertola AE Zunta-Soares G Swann AC . Does anxiety increase impulsivity in patients with bipolar disorder or major depressive disorder? J Psychiatr Res. (2012) 46:616–21. doi: 10.1016/j.jpsychires.2012.01.016 22326294

[88] Oreski I Jakovljevic M Aukst-Margetic B Orlic ZC Vuksan-Cusa B . Comorbidity and multimorbidity in patients with schizophrenia and bipolar disorder: similarities and differencies. Psychiatr Danub. (2012) 24:80–5. 22447090

[89] Moor S Crowe M Luty S Carter J Joyce PR . Effects of comorbidity and early age of onset in young people with Bipolar Disorder on self harming behaviour and suicide attempts. J Affect Disord. (2012) 136:1212–5. doi: 10.1016/j.jad.2011.10.018 22085804

[90] Young LT Cooke RG Robb JC Levitt AJ Joffe RT . Anxious and non-anxious bipolar disorder. J Affect Disord. (1993) 29:49–52. doi: 10.1016/0165-0327(93)90118-4 8254143

[91] Altshuler LL Kupka RW Hellemann G Frye MA Sugar CA McElroy SL . Gender and depressive symptoms in 711 patients with bipolar disorder evaluated prospectively in the Stanley Foundation bipolar treatment outcome network. Am J Psychiatry. (2010) 167:708–15. doi: 10.1176/appi.ajp.2009.09010105 20231325

[92] Azorin JM Kaladjian A Adida M Hantouche EG Hameg A Lancrenon S . Psychopathological correlates of lifetime anxiety comorbidity in bipolar I patients: findings from a French national cohort. Psychopathology. (2009) 42:380–6. doi: 10.1159/000241193 19776668

[93] Boylan KR Bieling PJ Marriott M Begin H Young LT MacQueen GM . Impact of comorbid anxiety disorders on outcome in a cohort of patients with bipolar disorder. J Clin Psychiatry. (2004) 65:1106–13. doi: 10.4088/jcp.v65n0813 15323597

[94] Ciapparelli A Paggini R Marazziti D Carmassi C Bianchi M Taponecco C . Comorbidity with axis I anxiety disorders in remitted psychotic patients 1 year after hospitalization. CNS Spectr. (2007) 12:913–9. doi: 10.1017/s1092852900015704 18163037

[95] Cosoff SJ Hafner RJ . The prevalence of comorbid anxiety in schizophrenia, schizoaffective disorder and bipolar disorder. Aust N Z J Psychiatry. (1998) 32:67–72. doi: 10.3109/00048679809062708 9565185

[96] Das A . Anxiety disorders in bipolar I mania: prevalence, effect on illness severity, and treatment implications. Indian J Psychol Med. (2013) 35:53–9. doi: 10.4103/0253-7176.112202 PMC3701361 23833343

[97] Freeman MP Freeman SA McElroy SL . The comorbidity of bipolar and anxiety disorders: prevalence, psychobiology, and treatment issues. J Affect Disord. (2002) 68:1–23. doi: 10.1016/s0165-0327(00)00299-8 11869778

[98] Goldstein BI Levitt AJ . The specific burden of comorbid anxiety disorders and of substance use disorders in bipolar I disorder. Bipolar Disord. (2008) 10:67–78. doi: 10.1111/j.1399-5618.2008.00461.x 18199243

[99] Henry C Van den Bulke D Bellivier F Etain B Rouillon F Leboyer M . Anxiety disorders in 318 bipolar patients: prevalence and impact on illness severity and response to mood stabilizer. J Clin Psychiatry. (2003) 64:331–5. doi: 10.4088/JCP.v64n0316 12716276

[100] Kawa I Carter JD Joyce PR Doughty CJ Frampton CM Wells JE . Gender differences in bipolar disorder: age of onset, course, comorbidity, and symptom presentation. Bipolar Disord. (2005) 7:119–25. doi: 10.1111/j.1399-5618.2004.00180.x 15762852

[101] Kessler RC Rubinow DR Holmes C Abelson JM Zhao S . The epidemiology of DSM-III-R bipolar I disorder in a general population survey. Psychol Med. (1997) 27:1079–89. doi: 10.1017/s0033291797005333 9300513

[102] Levander E Frye MA McElroy S Suppes T Grunze H Nolen WA . Alcoholism and anxiety in bipolar illness: differential lifetime anxiety comorbidity in bipolar I women with and without alcoholism. J Affect Disord. (2007) 101:211–7. doi: 10.1016/j.jad.2006.11.023 17254638

[103] Mantere O Isometsa E Ketokivi M Kiviruusu O Suominen K Valtonen HM . A prospective latent analyses study of psychiatric comorbidity of DSM-IV bipolar I and II disorders. Bipolar Disord. (2010) 12:271–84. doi: 10.1111/j.1399-5618.2010.00810.x 20565434

[104] Nakagawa A Grunebaum MF Sullivan GM Currier D Ellis SP Burke AK . Comorbid anxiety in bipolar disorder: does it have an independent effect on suicidality? Bipolar Disord. (2008) 10:530–8. doi: 10.1111/j.1399-5618.2008.00590.x PMC2746654 18452449

[105] Nery-Fernandes F Quarantini LC Galvao-De-Almeida A Rocha MV Kapczinski F Miranda-Scippa A . Lower rates of comorbidities in euthymic bipolar patients. World J Biol Psychiatry. (2009) 10:474–9. doi: 10.1080/15622970802688929 19401946

[106] Otto MW Simon NM Wisniewski SR Miklowitz DJ Kogan JN Reilly-Harrington NA . Prospective 12-month course of bipolar disorder in out-patients with and without comorbid anxiety disorders. Br J Psychiatry. (2006) 189:20–5. doi: 10.1192/bjp.bp.104.007773 16816301

[107] Schaffer A Cairney J Cheung A Veldhuizen S Levitt A . Community survey of bipolar disorder in Canada: lifetime prevalence and illness characteristics. Can J Psychiatry. (2006) 51:9–16. doi: 10.1177/070674370605100104 16491979

[108] Simon NM Otto MW Wisniewski SR Fossey M Sagduyu K Frank E . Anxiety disorder comorbidity in bipolar disorder patients: data from the first 500 participants in the Systematic Treatment Enhancement Program for Bipolar Disorder (STEP-BD). Am J Psychiatry. (2004) 161:2222–9. doi: 10.1176/appi.ajp.161.12.2222 15569893

[109] Szadoczky E Papp Z Vitrai J Rihmer Z Furedi J . The prevalence of major depressive and bipolar disorders in Hungary. Results from a national epidemiologic survey. J Affect Disord. (1998) 50:153–62. doi: 10.1016/s0165-0327(98)00056-1 9858075

[110] Tamam L Ozpoyraz N . Comorbidity of anxiety disorder among patients with bipolar I disorder in remission. Psychopathology. (2002) 35:203–9. doi: 10.1159/000063824 12239436

[111] Weber NS Fisher JA Cowan DN Niebuhr DW . Psychiatric and general medical conditions comorbid with bipolar disorder in the National Hospital Discharge Survey. Psychiatr Serv. (2011) 62:1152–8. doi: 10.1176/ps.62.10.pss6210_1152 21969641

[112] Young S Pfaff D Lewandowski KE Ravichandran C Cohen BM Ongur D . Anxiety disorder comorbidity in bipolar disorder, schizophrenia and schizoaffective disorder. Psychopathology. (2013) 46:176–85. doi: 10.1159/000339556 22906962

[113] Zutshi A Reddy YC Thennarasu K Chandrashekhar CR . Comorbidity of anxiety disorders in patients with remitted bipolar disorder. Eur Arch Psychiatry Clin Neurosci. (2006) 256:428–36. doi: 10.1007/s00406-006-0658-2 16783496

[114] Coryell W Solomon DA Fiedorowicz JG Endicott J Schettler PJ Judd LL . Anxiety and outcome in bipolar disorder. Am J Psychiatry. (2009) 166:1238–43. doi: 10.1176/appi.ajp.2009.09020218 PMC3551283 19797434

[115] Swann AC . The strong relationship between bipolar and substance-use disorder. Ann N Y Acad Sci. (2010) 1187:276–93. doi: 10.1111/j.1749-6632.2009.05146.x 20201858

[116] Regier DA Farmer ME Rae DS Locke BZ Keith SJ Judd LL . Comorbidity of mental disorders with alcohol and other drug abuse. Results from the Epidemiologic Catchment Area (ECA) Study. JAMA. (1990) 264:2511–8. doi: 10.1001/jama.1990.03450190043026 2232018

[117] Duffy A Horrocks J Milin R Doucette S Persson G Grof P . Adolescent substance use disorder during the early stages of bipolar disorder: a prospective high-risk study. J Affect Disord. (2012) 142:57–64. doi: 10.1016/j.jad.2012.04.010 22959686

[118] Cerullo MA Strakowski SM . The prevalence and significance of substance use disorders in bipolar type I and II disorder. Subst Abuse Treat Prev Policy. (2007) 2:29. doi: 10.1186/1747-597X-2-29 17908301 PMC2094705

[119] Lagerberg TV Andreassen OA Ringen PA Berg AO Larsson S Agartz I . Excessive substance use in bipolar disorder is associated with impaired functioning rather than clinical characteristics, a descriptive study. BMC Psychiatry. (2010) 10:9. doi: 10.1186/1471-244X-10-9 20105311 PMC2824653

[120] Di Florio A Craddock N van den Bree M . Alcohol misuse in bipolar disorder. A systematic review and meta-analysis of comorbidity rates. Eur Psychiatry. (2014) 29:117–24. doi: 10.1016/j.eurpsy.2013.07.004 24075633

[121] Estroff TW Dackis CA Gold MS Pottash AL . Drug abuse and bipolar disorders. Int J Psychiatry Med. (1985) 15:37–40. doi: 10.2190/6d4m-j23x-l21c-tp21 4055245

[122] Farren CK Hill KP Weiss RD . Bipolar disorder and alcohol use disorder: a review. Curr Psychiatry Rep. (2012) 14:659–66. doi: 10.1007/s11920-012-0320-9 PMC3730445 22983943

[123] Freed EX . Alcohol abuse by manic patients. Psychol Rep. (1969) 25:280. doi: 10.2466/pr0.1969.25.1.280 5366393

[124] Lai HM Sitharthan T Huang QR . Exploration of the comorbidity of alcohol use disorders and mental health disorders among inpatients presenting to all hospitals in New South Wales, Australia. Subst Abus. (2012) 33:138–45. doi: 10.1080/08897077.2011.634967 22489586

[125] Morrison JR . Bipolar affective disorder and alcoholism. Am J Psychiatry. (1974) 131:1130–3. doi: 10.1176/ajp.131.10.1130 4412212

[126] Goodwin F Jamison K . Manic-depressive illness. 2nd ed. New York: Oxford University Press (2007).

[127] Lai HM Sitharthan T . Exploration of the comorbidity of cannabis use disorders and mental health disorders among inpatients presenting to all hospitals in New South Wales, Australia. Am J Drug Alcohol Abuse. (2012) 38:567–74. doi: 10.3109/00952990.2012.694523 22746224

[128] Kennedy SH Welsh BR Fulton K Soczynska JK McIntyre RS O’Donovan C . Frequency and correlates of gambling problems in outpatients with major depressive disorder and bipolar disorder. Can J Psychiatry. (2010) 55:568–76. doi: 10.1177/070674371005500905 20840804

[129] McIntyre RS McElroy SL Konarski JZ Soczynska JK Wilkins K Kennedy SH . Problem gambling in bipolar disorder: results from the Canadian Community Health Survey. J Affect Disord. (2007) 102:27–34. doi: 10.1016/j.jad.2006.12.005 17240457

[130] Chengappa KN Levine J Gershon S Kupfer DJ . Lifetime prevalence of substance or alcohol abuse and dependence among subjects with bipolar I and II disorders in a voluntary registry. Bipolar Disord. (2000) 2:191–5. doi: 10.1034/j.1399-5618.2000.020306.x 11256686

[131] Weiss RD Mirin SM . Subtypes of cocaine abusers. Psychiatr Clin North Am. (1986) 9:491–501. doi: 10.1016/S0193-953X(18)30608-7 3774602

[132] Weiss RD Mirin SM Michael JL Sollogub AC . Psychopathology in chronic cocaine abusers. Am J Drug Alcohol Abuse. (1986) 12:17–29. doi: 10.3109/00952998609083740 3788897

[133] Miller FT Busch F Tanenbaum JH . Drug abuse in schizophrenia and bipolar disorder. Am J Drug Alcohol Abuse. (1989) 15:291–5. doi: 10.3109/00952998908993409 2763984

[134] Himmelhoch JM Mulla D Neil JF Detre TP Kupfer DJ . Incidence and signficiance of mixed affective states in a bipolar population. Arch Gen Psychiatry. (1976) 33:1062–6. doi: 10.1001/archpsyc.1976.01770090052004 962490

[135] Winokur G Clayton P Reich T . Manic Depressive Illness. Saint Louis: CV Mosby (1969).

[136] Goldberg JF Whiteside JE . The association between substance abuse and antidepressant-induced mania in bipolar disorder: a preliminary study. J Clin Psychiatry. (2002) 63:791–5. doi: 10.4088/jcp.v63n0907 12363119

[137] Magalhaes PV Kapczinski F Nierenberg AA Deckersbach T Weisinger D Dodd S . Illness burden and medical comorbidity in the Systematic Treatment Enhancement Program for Bipolar Disorder. Acta Psychiatr Scand. (2012) 125:303–8. doi: 10.1111/j.1600-0447.2011.01794.x 22098628

[138] McIntyre RS McElroy SL Konarski JZ Soczynska JK Bottas A Castel S . Substance use disorders and overweight/obesity in bipolar I disorder: preliminary evidence for competing addictions. J Clin Psychiatry. (2007) 68:1352–7. doi: 10.4088/jcp.v68n0905 17915973

[139] Meade CS Graff FS Griffin ML Weiss RD . HIV risk behavior among patients with co-occurring bipolar and substance use disorders: associations with mania and drug abuse. Drug Alcohol Depend. (2008) 92:296–300. doi: 10.1016/j.drugalcdep.2007.07.013 17850993 PMC2262843

[140] Goldstein BI Strober MA Birmaher B Axelson DA Esposito-Smythers C Goldstein TR . Substance use disorders among adolescents with bipolar spectrum disorders. Bipolar Disord. (2008) 10:469–78. doi: 10.1111/j.1399-5618.2008.00584.x PMC2768482 18452443

[141] Daban C Martinez-Aran A Torrent C Tabares-Seisdedos R Balanza-Martinez V Salazar-Fraile J . Specificity of cognitive deficits in bipolar disorder versus schizophrenia. A systematic review. Psychother Psychosom. (2006) 75:72–84. doi: 10.1159/000090891 16508342

[142] Goldberg JF Harrow M Grossman LS . Course and outcome in bipolar affective disorder: a longitudinal follow-up study. Am J Psychiatry. (1995) 152:379–84. doi: 10.1176/ajp.152.3.379 7864263

[143] Goldberg JF Harrow M Grossman LS . Recurrent affective syndromes in bipolar and unipolar mood disorders at follow-up. Br J Psychiatry. (1995) 166:382–5. doi: 10.1192/bjp.166.3.382 7788132

[144] Keck PE Jr. McElroy SL Strakowski SM West SA Sax KW Hawkins JM . 12-month outcome of patients with bipolar disorder following hospitalization for a manic or mixed episode. Am J Psychiatry. (1998) 155:646–52. doi: 10.1176/ajp.155.5.646 9585716

[145] Mur M Portella MJ Martinez-Aran A Pifarre J Vieta E . Persistent neuropsychological deficit in euthymic bipolar patients: executive function as a core deficit. J Clin Psychiatry. (2007) 68:1078–86. doi: 10.4088/jcp.v68n0715 17685745

[146] Strakowski SM Keck PE Jr. McElroy SL West SA Sax KW Hawkins JM . Twelve-month outcome after a first hospitalization for affective psychosis. Arch Gen Psychiatry. (1998) 55:49–55. doi: 10.1001/archpsyc.55.1.49 9435760

[147] Dore G Romans SE . Impact of bipolar affective disorder on family and partners. J Affect Disord. (2001) 67:147–58. doi: 10.1016/s0165-0327(01)00450-5 11869762

[148] Huxley N Baldessarini RJ . Disability and its treatment in bipolar disorder patients. Bipolar Disord. (2007) 9:183–96. doi: 10.1111/j.1399-5618.2007.00430.x 17391360

[149] Murray CJL Lopez AD eds. The global burden of disease. A comprehensive assessment of the mortality and disability from diseases, injuries and risk factors in 1990 and projected to 2020. Boston, MA: Harvard School of Public Health on behalf of the World Health Organisation, and the World Bank (1996).

[150] Pope M Dudley R Scott J . Determinants of social functioning in bipolar disorder. Bipolar Disord. (2007) 9:38–44. doi: 10.1111/j.1399-5618.2007.00323.x 17391348

[151] Simon GE . Social and economic burden of mood disorders. Biol Psychiatry. (2003) 54:208–15. doi: 10.1016/s0006-3223(03)00420-7 12893097

[152] Gutierrez-Rojas L Jurado D Gurpegui M . Factors associated with work, social life and family life disability in bipolar disorder patients. Psychiatry Res. (2011) 186:254–60. doi: 10.1016/j.psychres.2010.06.020 20647154

[153] Judd LL Schettler PJ Solomon DA Maser JD Coryell W Endicott J . Psychosocial disability and work role function compared across the long-term course of bipolar I, bipolar II and unipolar major depressive disorders. J Affect Disord. (2008) 108:49–58. doi: 10.1016/j.jad.2007.06.014 18006071

[154] Bauer M Glenn T Grof P Rasgon NL Marsh W Sagduyu K . Frequency of subsyndromal symptoms and employment status in patients with bipolar disorder. Soc Psychiatry Psychiatr Epidemiol. (2009) 44:515–22. doi: 10.1007/s00127-008-0464-4 19011720

[155] Bowden CL Krishnan AA . Pharmacotherapy for bipolar depression: an economic assessment. Expert Opin Pharmacother. (2004) 5:1101–7. doi: 10.1517/14656566.5.5.1101 15155111

[156] Bowie CR Depp C McGrath JA Wolyniec P Mausbach BT Thornquist MH . Prediction of real-world functional disability in chronic mental disorders: a comparison of schizophrenia and bipolar disorder. Am J Psychiatry. (2010) 167:1116–24. doi: 10.1176/appi.ajp.2010.09101406 PMC3694770 20478878

[157] Judd LL Akiskal HS Schettler PJ Endicott J Leon AC Solomon DA . Psychosocial disability in the course of bipolar I and II disorders: a prospective, comparative, longitudinal study. Arch Gen Psychiatry. (2005) 62:1322–30. doi: 10.1001/archpsyc.62.12.1322 16330720

[158] Kauer-Sant’Anna M Bond DJ Lam RW Yatham LN . Functional outcomes in first-episode patients with bipolar disorder: a prospective study from the Systematic Treatment Optimization Program for Early Mania project. Compr Psychiatry. (2009) 50:1–8. doi: 10.1016/j.comppsych.2008.05.013 19059506

[159] Marangell LB . The importance of subsyndromal symptoms in bipolar disorder. J Clin Psychiatry. (2004) 65 Suppl 10:24–7. 15242329

[160] Rosa AR Reinares M Michalak EE Bonnin CM Sole B Franco C . Functional impairment and disability across mood states in bipolar disorder. Value Health. (2010) 13:984–8. doi: 10.1111/j.1524-4733.2010.00768.x 20667057

[161] Sanchez-Moreno J Martinez-Aran A Tabares-Seisdedos R Torrent C Vieta E Ayuso-Mateos JL . Functioning and disability in bipolar disorder: an extensive review. Psychother Psychosom. (2009) 78:285–97. doi: 10.1159/000228249 19602917

[162] Simon GE Bauer MS Ludman EJ Operskalski BH Unutzer J . Mood symptoms, functional impairment, and disability in people with bipolar disorder: specific effects of mania and depression. J Clin Psychiatry. (2007) 68:1237–45. doi: 10.4088/jcp.v68n0811 17854249

[163] Wingo AP Baldessarini RJ Compton MT Harvey PD . Correlates of recovery of social functioning in types I and II bipolar disorder patients. Psychiatry Res. (2010) 177:131–4. doi: 10.1016/j.psychres.2010.02.020 PMC2859974 20334933

[164] Depp CA Davis CE Mittal D Patterson TL Jeste DV . Health-related quality of life and functioning of middle-aged and elderly adults with bipolar disorder. J Clin Psychiatry. (2006) 67:215–21. doi: 10.4088/jcp.v67n0207 16566616

[165] Michalak EE Murray G Young AH Lam RW . Burden of bipolar depression: impact of disorder and medications on quality of life. CNS Drugs. (2008) 22:389–406. doi: 10.2165/00023210-200822050-00003 18399708

[166] Ozer S Ulusahin A Batur S Kabakci E Saka MC . Outcome measures of interepisode bipolar patients in a Turkish sample. Soc Psychiatry Psychiatr Epidemiol. (2002) 37:31–7. doi: 10.1007/s127-002-8211-z 11924747

[167] Depp CA Mausbach BT Eyler LT Palmer BW Cain AE Lebowitz BD . Performance-based and subjective measures of functioning in middle-aged and older adults with bipolar disorder. J Nerv Ment Dis. (2009) 197:471–5. doi: 10.1097/NMD.0b013e3181ab5c9b 19597353

[168] Chand PK Mattoo SK Sharan P . Quality of life and its correlates in patients with bipolar disorder stabilized on lithium prophylaxis. Psychiatry Clin Neurosci. (2004) 58:311–8. doi: 10.1111/j.1440-1819.2004.01237.x 15149299

[169] Brodaty H Green A . Who cares for the carer? The often forgotten patient. Aust Fam Physician. (2002) 31:833–6. 12402702

[170] Lowery K Mynt P Aisbett J Dixon T O’Brien J Ballard C . Depression in the carers of dementia sufferers: a comparison of the carers of patients suffering from dementia with Lewy bodies and the carers of patients with Alzheimer’s disease. J Affect Disord. (2000) 59:61–5. doi: 10.1016/s0165-0327(99)00123-8 10814772

[171] Platt S . Measuring the burden of psychiatric illness on the family: an evaluation of some rating scales. Psychol Med. (1985) 15:383–93. doi: 10.1017/s0033291700023680 4023142

[172] Fadden G Bebbington P Kuipers L . The burden of care: the impact of functional psychiatric illness on the patient’s family. Br J Psychiatry. (1987) 150:285–92. doi: 10.1192/bjp.150.3.285 3311267

[173] Grad J Sainbury M . Evaluating a community care service. In: Freeman H Farndale J , editors. Trends in mental health services. Pergamon Press, Oxford (1963).

[174] Grad J Sainsbury P . Mental illness and the family. Lancet. (1963) 1:544–7. doi: 10.1016/s0140-6736(63)91339-4 13949711

[175] Hoenig J Hamilton M . The desegregation of the mentally ill. London: Routledge and Keegan-Paul (1969).

[176] Hoenig J Hamilton MW . The schizophrenic patient in the community and his effect on the household. Int J Soc Psychiatry. (1966) 12:165–76. doi: 10.1177/002076406601200301 5964677

[177] Chakrabarti S Gill S . Coping and its correlates among caregivers of patients with bipolar disorder: a preliminary study. Bipolar Disord. (2002) 4:50–60. doi: 10.1034/j.1399-5618.2002.01167.x 12047495

[178] Cook JA Lefley HP Pickett SA Cohler BJ . Age and family burden among parents of offspring with severe mental illness. Am J Orthopsychiatry. (1994) 64:435–47. doi: 10.1037/h0079535 7977666

[179] Maji KR Sood M Sagar R Khandelwal SK . A follow-up study of family burden in patients with bipolar affective disorder. Int J Soc Psychiatry. (2012) 58:217–23. doi: 10.1177/0020764010390442 21421638

[180] Perlick D Clarkin JF Sirey J Raue P Greenfield S Struening E . Burden experienced by care-givers of persons with bipolar affective disorder. Br J Psychiatry. (1999) 175:56–62. doi: 10.1192/bjp.175.1.56 10621769

[181] Perlick DA Rosenheck RA Miklowitz DJ Chessick C Wolff N Kaczynski R . Prevalence and correlates of burden among caregivers of patients with bipolar disorder enrolled in the Systematic Treatment Enhancement Program for Bipolar Disorder. Bipolar Disord. (2007) 9:262–73. doi: 10.1111/j.1399-5618.2007.00365.x 17430301

[182] Chakrabarti S Kulhara P Verma SK . Extent and determinants of burden among families of patients with affective disorders. Acta Psychiatr Scand. (1992) 86:247–52. doi: 10.1111/j.1600-0447.1992.tb03261.x 1414422

[183] Amore M Menchetti M Tonti C Scarlatti F Lundgren E Esposito W . Predictors of violent behavior among acute psychiatric patients: clinical study. Psychiatry Clin Neurosci. (2008) 62:247–55. doi: 10.1111/j.1440-1819.2008.01790.x 18588583

[184] Currier GW Allen MH . Emergency psychiatry: physical and chemical restraint in the psychiatric emergency service. Psychiatr Serv. (2000) 51:717–9. doi: 10.1176/appi.ps.51.6.717 10828101

[185] Raveendranathan D Chandra PS Chaturvedi SK . Violence among psychiatric inpatients: a victim’s perspective. East Asian Arch Psychiatry. (2012) 22:141–5. 23271582

[186] Targum SD Dibble ED Davenport YB Gershon ES . The Family Attitudes Questionnaire. Patients’ and spouses’ views of bipolar illness. Arch Gen Psychiatry. (1981) 38:562–8. doi: 10.1001/archpsyc.1980.01780300074009 7235858

[187] Eisdorfer C . Caregiving: an emerging risk factor for emotional and physical pathology. Bull Menninger Clin. (1991) 55:238–47. 2043900

[188] Gallagher SK Mechanic D . Living with the mentally ill: effects on the health and functioning of other household members. Soc Sci Med. (1996) 42:1691–701. doi: 10.1016/0277-9536(95)00296-0 8783431

[189] Iacovides A Fountoulakis K Moysidou C Ierodiakonou C . Burnout in nursing staff: a clinical syndrome rather than a psychological reaction? Gen Hosp Psychiatry. (1997) 19:419–28. 10.1016/s0163-8343(97)00070-4 9438186

[190] Perlick DA Hohenstein JM Clarkin JF Kaczynski R Rosenheck RA . Use of mental health and primary care services by caregivers of patients with bipolar disorder: a preliminary study. Bipolar Disord. (2005) 7:126–35. doi: 10.1111/j.1399-5618.2004.00172.x 15762853

[191] Perlick DA Rosenheck RA Miklowitz DJ Kaczynski R Link B Ketter T . Caregiver burden and health in bipolar disorder: a cluster analytic approach. J Nerv Ment Dis. (2008) 196:484–91. doi: 10.1097/NMD.0b013e3181773927 PMC4006696 18552626

[192] Steele A Maruyama N Galynker I . Psychiatric symptoms in caregivers of patients with bipolar disorder: a review. J Affect Disord. (2010) 121:10–21. doi: 10.1016/j.jad.2009.04.020 19443040

[193] Angst J Angst F Gerber-Werder R Gamma A . Suicide in 406 mood-disorder patients with and without long-term medication: a 40 to 44 years’ follow-up. Arch Suicide Res. (2005) 9:279–300. doi: 10.1080/13811110590929488 16020171

[194] Goodwin FK Jamison KR Goodwin F . Suicide. In: Goodwin FK Jamison KR , editors. Manic-depressive illness. Oxford University Press, New York (1990). p. 227–44.

[195] Guze SB Robins E . Suicide and primary affective disorders. Br J Psychiatry. (1970) 117:437–8. doi: 10.1192/bjp.117.539.437 5481206

[196] Harris EC Barraclough B . Suicide as an outcome for mental disorders. A meta-analysis. Br J Psychiatry. (1997) 170:205–28. doi: 10.1192/bjp.170.3.205 9229027

[197] Elizabeth Sublette M Carballo JJ Moreno C Galfalvy HC Brent DA Birmaher B . Substance use disorders and suicide attempts in bipolar subtypes. J Psychiatr Res. (2009) 43:230–8. doi: 10.1016/j.jpsychires.2008.05.001 PMC2671238 18590916

[198] Oquendo MA Currier D Liu SM Hasin DS Grant BF Blanco C . Increased risk for suicidal behavior in comorbid bipolar disorder and alcohol use disorders: results from the National Epidemiologic Survey on Alcohol and Related Conditions (NESARC). J Clin Psychiatry. (2010) 71:902–9. doi: 10.4088/JCP.09m05198gry PMC2914308 20667292

[199] McCarthy JF Valenstein M Zivin K Zeber JE Kilbourne AM . Access-related measures and out-of-system utilization among veterans with bipolar disorder. Psychiatr Serv. (2010) 61:1035–8. doi: 10.1176/ps.2010.61.10.1035 20889645

[200] Centorrino F Mark TL Talamo A Oh K Chang J . Health and economic burden of metabolic comorbidity among individuals with bipolar disorder. J Clin Psychopharmacol. (2009) 29:595–600. doi: 10.1097/JCP.0b013e3181bef8a6 19910727

[201] Chisholm D van Ommeren M Ayuso-Mateos JL Saxena S . Cost-effectiveness of clinical interventions for reducing the global burden of bipolar disorder. Br J Psychiatry. (2005) 187:559–67. doi: 10.1192/bjp.187.6.559 16319409

[202] Das Gupta R Guest JF . Annual cost of bipolar disorder to UK society. Br J Psychiatry. (2002) 180:227–33. doi: 10.1192/bjp.180.3.227 11872515

[203] de Zelicourt M Dardennes R Verdoux H Gandhi G Khoshnood B Chomette E . Frequency of hospitalisations and inpatient care costs of manic episodes: in patients with bipolar I disorder in France. Pharmacoeconomics. (2003) 21:1081–90. doi: 10.2165/00019053-200321150-00002 14596627

[204] Dilsaver SC . An estimate of the minimum economic burden of bipolar I and II disorders in the United States: 2009. J Affect Disord. (2011) 129:79–83. doi: 10.1016/j.jad.2010.08.030 20888048

[205] Fajutrao L Locklear J Priaulx J Heyes A . A systematic review of the evidence of the burden of bipolar disorder in Europe. Clin Pract Epidemiol Ment Health. (2009) 5:3. doi: 10.1186/1745-0179-5-3 19166608 PMC2646705

[206] Olie JP Levy E . Manic episodes: the direct cost of a three-month period following hospitalisation. Eur Psychiatry. (2002) 17:278–86. doi: 10.1016/s0924-9338(02)00680-6 12381498

[207] Brook RA Rajagopalan K Kleinman NL Smeeding JE Brizee TJ Gardner HH . Incurring greater health care costs: risk stratification of employees with bipolar disorder. Prim Care Companion J Clin Psychiatry. (2006) 8:17–24. doi: 10.4088/pcc.v08n0103 16862249 PMC1510906

[208] Peele PB Xu Y Kupfer DJ . Insurance expenditures on bipolar disorder: clinical and parity implications. Am J Psychiatry. (2003) 160:1286–90. doi: 10.1176/appi.ajp.160.7.1286 12832243

[209] Stender M Bryant-Comstock L Phillips S . Medical resource use among patients treated for bipolar disorder: a retrospective, cross-sectional, descriptive analysis. Clin Ther. (2002) 24:1668–76. doi: 10.1016/s0149-2918(02)80070-4 12462295

[210] Merikangas KR Jin R He JP Kessler RC Lee S Sampson NA . Prevalence and correlates of bipolar spectrum disorder in the world mental health survey initiative. Arch Gen Psychiatry. (2011) 68:241–51. doi: 10.1001/archgenpsychiatry.2011.12 PMC3486639 21383262

[211] Kilbourne AM Perron BE Mezuk B Welsh D Ilgen M Bauer MS . Co-occurring conditions and health-related quality of life in patients with bipolar disorder. Psychosom Med. (2009) 71:894–900. doi: 10.1097/PSY.0b013e3181b49948 19661187

[212] McIntyre RS Konarski JZ Soczynska JK Wilkins K Panjwani G Bouffard B . Medical comorbidity in bipolar disorder: implications for functional outcomes and health service utilization. Psychiatr Serv. (2006) 57:1140–4. doi: 10.1176/ps.2006.57.8.1140 16870965

[213] Soreca I Frank E Kupfer DJ . The phenomenology of bipolar disorder: what drives the high rate of medical burden and determines long-term prognosis? Depress Anxiety. (2009) 26:73–82. doi: 10.1002/da.20521 18828143 PMC3308337

[214] Bryant-Comstock L Stender M Devercelli G . Health care utilization and costs among privately insured patients with bipolar I disorder. Bipolar Disord. (2002) 4:398–405. doi: 10.1034/j.1399-5618.2002.01148.x 12519100

[215] Gardner HH Kleinman NL Brook RA Rajagopalan K Brizee TJ Smeeding JE . The economic impact of bipolar disorder in an employed population from an employer perspective. J Clin Psychiatry. (2006) 67:1209–18. doi: 10.4088/jcp.v67n0806 16965198

[216] Alstrom C . Mortality in mental hospitals. Acta Psychiatr Neurol. (1942) 17:1–42.

[217] Babigian HM Odoroff CL . The mortality experience of a population with psychiatric illness. Am J Psychiatry. (1969) 126:470–80. doi: 10.1176/ajp.126.4.470 5806790

[218] Fagiolini A Goracci A . The effects of undertreated chronic medical illnesses in patients with severe mental disorders. J Clin Psychiatry. (2009) 70 Suppl 3:22–9. doi: 10.4088/JCP.7075su1c.04 19570498

[219] Colton CW Manderscheid RW . Congruencies in increased mortality rates, years of potential life lost, and causes of death among public mental health clients in eight states. Prev Chronic Dis. (2006) 3:A42. 16539783 PMC1563985

[220] Beyer J Kuchibhatla M Gersing K Krishnan KR . Medical comorbidity in a bipolar outpatient clinical population. Neuropsychopharmacology. (2005) 30:401–4. doi: 10.1038/sj.npp.1300608 15536492

[221] Carney CP Jones LE . Medical comorbidity in women and men with bipolar disorders: a population-based controlled study. Psychosom Med. (2006) 68:684–91. doi: 10.1097/01.psy.0000237316.09601.88 17012521

[222] Castelo MS Hyphantis TN Macedo DS Lemos GO MaChado YO Kapczinski F . Screening for bipolar disorder in the primary care: a Brazilian survey. J Affect Disord. (2012) 143:118–24. doi: 10.1016/j.jad.2012.05.040 22959680

[223] Douzenis A Seretis D Nika S Nikolaidou P Papadopoulou A Rizos EN . Factors affecting hospital stay in psychiatric patients: the role of active comorbidity. BMC Health Serv Res. (2012) 12:166. doi: 10.1186/1472-6963-12-166 22713232 PMC3470970

[224] Feldman NS Gwizdowski IS Fischer EG Yang H Suppes T . Co-occurrence of serious or undiagnosed medical conditions with bipolar disorder preventing clinical trial randomization: a case series. J Clin Psychiatry. (2012) 73:874–7. doi: 10.4088/JCP.11m07331 22480536

[225] Kemp DE Sylvia LG Calabrese JR Nierenberg AA Thase ME Reilly-Harrington NA . General medical burden in bipolar disorder: findings from the LiTMUS comparative effectiveness trial. Acta Psychiatr Scand. (2014) 129:24–34. doi: 10.1111/acps.12101 23465084 PMC3789858

[226] Kilbourne AM Cornelius JR Han X Pincus HA Shad M Salloum I . Burden of general medical conditions among individuals with bipolar disorder. Bipolar Disord. (2004) 6:368–73. doi: 10.1111/j.1399-5618.2004.00138.x 15383128

[227] McIntyre RS Soczynska JK Beyer JL Woldeyohannes HO Law CW Miranda A . Medical comorbidity in bipolar disorder: re-prioritizing unmet needs. Curr Opin Psychiatry. (2007) 20:406–16. doi: 10.1097/YCO.0b013e3281938102 17551358

[228] Perron BE Howard MO Nienhuis JK Bauer MS Woodward AT Kilbourne AM . Prevalence and burden of general medical conditions among adults with bipolar I disorder: results from the National Epidemiologic Survey on Alcohol and Related Conditions. J Clin Psychiatry. (2009) 70:1407–15. doi: 10.4088/JCP.08m04586yel 19906344

[229] Strakowski SM MeElroy SL Keck PW Jr. West SA . The co-occurrence of mania with medical and other psychiatric disorders. Int J Psychiatry Med. (1994) 24:305–28. doi: 10.2190/CM8E-46R5-9AJL-03FN 7737787

[230] Fountoulakis K . Staging of bipolar disorder. In: Fountoulakis K , editor. Bipolar Disorder: An Evidence-Based Guide to Manic Depression. Springer-Verlag Berlin Heidelberg (2015). p. 437–59.

[231] Berk M Conus P Lucas N Hallam K Malhi GS Dodd S . Setting the stage: from prodrome to treatment resistance in bipolar disorder. Bipolar Disord. (2007) 9:671–8. doi: 10.1111/j.1399-5618.2007.00484.x 17988356

[232] Berk M Hallam KT McGorry PD . The potential utility of a staging model as a course specifier: a bipolar disorder perspective. J Affect Disord. (2007) 100:279–81. doi: 10.1016/j.jad.2007.03.007 17433450

[233] Cosci F Fava GA . Staging of mental disorders: systematic review. Psychother Psychosom. (2013) 82:20–34. doi: 10.1159/000342243 23147126

[234] Frank E Nimgaonkar VL Phillips ML Kupfer DJ . All the world’s a (clinical) stage: rethinking bipolar disorder from a longitudinal perspective. Mol Psychiatry. (2015) 20:23–31. doi: 10.1038/mp.2014.71 25048003 PMC4303542

[235] Kapczinski F Dias VV Kauer-Sant’Anna M Frey BN Grassi-Oliveira R Colom F . Clinical implications of a staging model for bipolar disorders. Expert Rev Neurother. (2009) 9:957–66. doi: 10.1586/ern.09.31 19589046

[236] Post RM . Mechanisms of illness progression in the recurrent affective disorders. Neurotox Res. (2010) 18:256–71. doi: 10.1007/s12640-010-9182-2 20390474

[237] Post RM Fleming J Kapczinski F . Neurobiological correlates of illness progression in the recurrent affective disorders. J Psychiatr Res. (2012) 46:561–73. doi: 10.1016/j.jpsychires.2012.02.004 22444599

[238] Berk M Berk L Udina M Moylan S Stafford L Hallam K . Palliative models of care for later stages of mental disorder: maximizing recovery, maintaining hope, and building morale. Aust N Z J Psychiatry. (2012) 46:92–9. doi: 10.1177/0004867411432072 22311525

[239] Torrent C Bonnin Cdel M Martinez-Aran A Valle J Amann BL Gonzalez-Pinto A . Efficacy of functional remediation in bipolar disorder: a multicenter randomized controlled study. Am J Psychiatry. (2013) 170:852–9. doi: 10.1176/appi.ajp.2012.12070971 23511717

[240] Fountoulakis KN . The contemporary face of bipolar illness: complex diagnostic and therapeutic challenges. CNS Spectr. (2008) 13:763–774, 777-769. doi: 10.1017/s1092852900013894 18849896

[241] Fountoulakis K . Special issues. In: Fountoulakis K , editor. Bipolar Disorder: An Evidence-Based Guide to Manic Depression. Springer-Verlag Berlin Heidelberg (2015). p. 659–84.

[242] Vrublevska J Fountoulakis K . Medical comorbidity in bipolar disorder. In: Yildiz A Ruiz P Nemeroff C , editors. The Bipolar Book. History, Neurobiology, and Treatment. Oxford University Press (2015). p. 497–506.

[243] Fountoulakis KN Gonda X Vieta E Rihmer Z . Class effect of pharmacotherapy in bipolar disorder: fact or misbelief? Ann Gen Psychiatry. (2011) 10:8. doi: 10.1186/1744-859X-10-8 21435226 PMC3078905

[244] Fountoulakis KN Vieta E Sanchez-Moreno J Kaprinis SG Goikolea JM Kaprinis GS . Treatment guidelines for bipolar disorder: a critical review. J Affect Disord. (2005) 86:1–10. doi: 10.1016/j.jad.2005.01.004 15820265

[245] Reinares M Rosa AR Franco C Goikolea JM Fountoulakis K Siamouli M . A systematic review on the role of anticonvulsants in the treatment of acute bipolar depression. Int J Neuropsychopharmacol. (2013) 16:485–96. doi: 10.1017/S1461145712000491 22575611

[246] Rosa A Fountoulakis K Siamouli M Gonda X Vieta E . Is anticonvulsant treatment of mania a class effect? Data from randomized clinical trials. CNS Neurosci Ther. (2011) 17:167–77. doi: 10.1111/j.1755-5949.2009.00089.x PMC6493801 20015083

[247] Fountoulakis KN Kelsoe JR Akiskal H . Receptor targets for antidepressant therapy in bipolar disorder: an overview. J Affect Disord. (2012) 138:222–38. doi: 10.1016/j.jad.2011.04.043 21601292

[248] Popovic D Reinares M Goikolea JM Bonnin CM Gonzalez-Pinto A Vieta E . Polarity index of pharmacological agents used for maintenance treatment of bipolar disorder. Eur Neuropsychopharmacol. (2012) 22:339–46. doi: 10.1016/j.euroneuro.2011.09.008 22000157

[249] Fountoulakis KN Kontis D Gonda X Siamouli M Yatham LN . Treatment of mixed bipolar states. Int J Neuropsychopharmacol. (2012) 15:1015–26. doi: 10.1017/S1461145711001817 22217434

[250] Fountoulakis KN Kontis D Gonda X Yatham LN . A systematic review of the evidence on the treatment of rapid cycling bipolar disorder. Bipolar Disord. (2013) 15:115–37. doi: 10.1111/bdi.12045 23437958

[251] Nivoli AM Colom F Murru A Pacchiarotti I Castro-Loli P Gonzalez-Pinto A . New treatment guidelines for acute bipolar depression: a systematic review. J Affect Disord. (2011) 129:14–26. doi: 10.1016/j.jad.2010.05.018 20538341

[252] Judd LL Akiskal HS Schettler PJ Endicott J Maser J Solomon DA . The long-term natural history of the weekly symptomatic status of bipolar I disorder. Arch Gen Psychiatry. (2002) 59:530–7. doi: 10.1001/archpsyc.59.6.530 12044195

[253] Morgan VA Mitchell PB Jablensky AV . The epidemiology of bipolar disorder: sociodemographic, disability and service utilization data from the Australian National Study of Low Prevalence (Psychotic) Disorders. Bipolar Disord. (2005) 7:326–37. doi: 10.1111/j.1399-5618.2005.00229.x 16026485

[254] Grande I Berk M Birmaher B Vieta E . Bipolar disorder. Lancet. (2016) 387:1561–72. doi: 10.1016/S0140-6736(15)00241-X 26388529

[255] Fountoulakis K . Biological therapies. In: Fountoulakis K , editor. Bipolar Disorder: An Evidence-Based Guide to Manic Depression. Springer-Verlag Berlin Heidelberg (2015). p. 461–625.

[256] Fountoulakis K . Psychosocial treatment and interventions. In: Fountoulakis K , editor. Bipolar Disorder: An Evidence-Based Guide to Manic Depression. Springer-Verlag Berlin Heidelberg (2015). p. 627–42.

[257] Fountoulakis KN Bech P Panagiotidis P Siamouli M Kantartzis S Papadopoulou A . Comparison of depressive indices: reliability, validity, relationship to anxiety and personality and the role of age and life events. J Affect Disord. (2007) 97:187–95. doi: 10.1016/j.jad.2006.06.015 16844229

[258] Fountoulakis KN Grunze H Panagiotidis P Kaprinis G . Treatment of bipolar depression: an update. J Affect Disord. (2008) 109:21–34. doi: 10.1016/j.jad.2007.10.016 18037498

[259] Fountoulakis KN Vieta E Siamouli M Valenti M Magiria S Oral T . Treatment of bipolar disorder: a complex treatment for a multi-faceted disorder. Ann Gen Psychiatry. (2007) 6:27. doi: 10.1186/1744-859X-6-27 17925035 PMC2089060

[260] Gonda X Fountoulakis KN Rihmer Z Lazary J Laszik A Akiskal KK . Towards a genetically validated new affective temperament scale: a delineation of the temperament phenotype of 5-HTTLPR using the TEMPS-A. J Affect Disord. (2009) 112:19–29. doi: 10.1016/j.jad.2008.03.012 18455241

[261] Pacchiarotti I Bond DJ Baldessarini RJ Nolen WA Grunze H Licht RW . The International Society for Bipolar Disorders (ISBD) task force report on antidepressant use in bipolar disorders. Am J Psychiatry. (2013) 170:1249–62. doi: 10.1176/appi.ajp.2013.13020185 PMC4091043 24030475

[262] Reinares M Sanchez-Moreno J Fountoulakis KN . Psychosocial interventions in bipolar disorder: what, for whom, and when. J Affect Disord. (2014) 156:46–55. doi: 10.1016/j.jad.2013.12.017 24439829

[263] Gerson LD Rose LE . Needs of persons with serious mental illness following discharge from inpatient treatment: patient and family views. Arch Psychiatr Nurs. (2012) 26:261–71. doi: 10.1016/j.apnu.2012.02.002 22835746

[264] Charney DS Reynolds CF 3rd Lewis L Lebowitz BD Sunderland T Alexopoulos GS . Depression and Bipolar Support Alliance consensus statement on the unmet needs in diagnosis and treatment of mood disorders in late life. Arch Gen Psychiatry. (2003) 60:664–72. doi: 10.1001/archpsyc.60.7.664 12860770

[265] Muralidharan K Ali M Silveira LE Bond DJ Fountoulakis KN Lam RW . Efficacy of second generation antipsychotics in treating acute mixed episodes in bipolar disorder: a meta-analysis of placebo-controlled trials. J Affect Disord. (2013) 150:408–14. doi: 10.1016/j.jad.2013.04.032 23735211

[266] Yatham LN Fountoulakis KN Rahman Z Ammerman D Fyans P Marler SV . Efficacy of aripiprazole versus placebo as adjuncts to lithium or valproate in relapse prevention of manic or mixed episodes in bipolar I patients stratified by index manic or mixed episode. J Affect Disord. (2013) 147:365–72. doi: 10.1016/j.jad.2012.11.042 23290791

[267] Curtis V . Women are not the same as men: specific clinical issues for female patients with bipolar disorder. Bipolar Disord. (2005) 7 Suppl 1:16–24. doi: 10.1111/j.1399-5618.2005.00190.x 15762865

[268] Hendrick V Altshuler LL Gitlin MJ Delrahim S Hammen C . Gender and bipolar illness. J Clin Psychiatry. (2000) 61:393–396; quiz 397. doi: 10.4088/jcp.v61n0514 10847318

[269] Leibenluft E . Women with bipolar illness: clinical and research issues. Am J Psychiatry. (1996) 153:163–73. doi: 10.1176/ajp.153.2.163 8561195

[270] Leibenluft E . Women and bipolar disorder: an update. Bull Menninger Clin. (2000) 64:5–17. 10695156

[271] Arnold LM McElroy SL Keck PE Jr . The role of gender in mixed mania. Compr Psychiatry. (2000) 41:83–7. doi: 10.1016/s0010-440x(00)90137-8 10741883

[272] McElroy SL Keck PE Jr. Pope HG Jr. Hudson JI Faedda GL Swann AC . Clinical and research implications of the diagnosis of dysphoric or mixed mania or hypomania. Am J Psychiatry. (1992) 149:1633–44. doi: 10.1176/ajp.149.12.1633 1359799

[273] Post RM Denicoff KD Leverich GS Altshuler LL Frye MA Suppes TM . Morbidity in 258 bipolar outpatients followed for 1 year with daily prospective ratings on the NIMH life chart method. J Clin Psychiatry. (2003) 64:680–690; quiz 738-689. doi: 10.4088/jcp.v64n0610 12823083

[274] Fakhoury WK Wright D Wallace M . Prevalence and extent of distress of adverse effects of antipsychotics among callers to a United Kingdom National Mental Health Helpline. Int Clin Psychopharmacol. (2001) 16:153–62. doi: 10.1097/00004850-200105000-00004 11354237

[275] Russell JM Mackell JA . Bodyweight gain associated with atypical antipsychotics: epidemiology and therapeutic implications. CNS Drugs. (2001) 15:537–51. doi: 10.2165/00023210-200115070-00004 11510624

[276] McElroy SL Frye MA Suppes T Dhavale D Keck PE Jr. Leverich GS . Correlates of overweight and obesity in 644 patients with bipolar disorder. J Clin Psychiatry. (2002) 63:207–13. doi: 10.4088/jcp.v63n0306 11926719

[277] Wieck A Haddad PM . Antipsychotic-induced hyperprolactinaemia in women: pathophysiology, severity and consequences. Selective literature review. Br J Psychiatry. (2003) 182:199–204. doi: 10.1192/bjp.182.3.199 12611781

[278] Smith S Wheeler MJ Murray R O’Keane V . The effects of antipsychotic-induced hyperprolactinaemia on the hypothalamic-pituitary-gonadal axis. J Clin Psychopharmacol. (2002) 22:109–14. doi: 10.1097/00004714-200204000-00002 11910254

[279] Coverdale JH Turbott SH Roberts H . Family planning needs and STD risk behaviours of female psychiatric out-patients. Br J Psychiatry. (1997) 171:69–72. doi: 10.1192/bjp.171.1.69 9328499

[280] Cohen LS Friedman JM Jefferson JW Johnson EM Weiner ML . A reevaluation of risk of in *utero* exposure to lithium. JAMA. (1994) 271:146–50. doi: 10.1001/jama.1994.03510260078033 8031346

[281] Packer S . Family planning for women with bipolar disorder. Hosp Community Psychiatry. (1992) 43:479–82. doi: 10.1176/ps.43.5.479 1587511

[282] Blehar MC DePaulo JR Jr. Gershon ES Reich T Simpson SG Nurnberger JI Jr . Women with bipolar disorder: findings from the NIMH Genetics Initiative sample. Psychopharmacol Bull. (1998) 34:239–43. 9803748

[283] Brockington IF Cernik KF Schofield EM Downing AR Francis AF Keelan C . Puerperal psychosis. Phenomena and diagnosis. Arch Gen Psychiatry. (1981) 38:829–33. doi: 10.1001/archpsyc.1981.01780320109013 7247645

[284] Davidson J Robertson E . A follow-up study of post partum illness 1946-1978. Acta Psychiatr Scand. (1985) 71:451–7. doi: 10.1111/j.1600-0447.1985.tb05057.x 4013805

[285] Dunner DL Patrick V Fieve RR . Life events at the onset of bipolar affective illness. Am J Psychiatry. (1979) 136:508–11. doi: 10.1176/ajp.1979.136.4b.508 426132

[286] Freeman MP Smith KW Freeman SA McElroy SL Kmetz GE Wright R . The impact of reproductive events on the course of bipolar disorder in women. J Clin Psychiatry. (2002) 63:284–7. doi: 10.4088/jcp.v63n0403 12004800

[287] Kendell RE Chalmers JC Platz C . Epidemiology of puerperal psychoses. Br J Psychiatry. (1987) 150:662–73. doi: 10.1192/bjp.150.5.662 3651704

[288] Schopf J Rust B . Follow-up and family study of postpartum psychoses. Part I: Overview. Eur Arch Psychiatry Clin Neurosci. (1994) 244:101–11. doi: 10.1007/BF02193527 7948053

[289] Chengappa KR Williams P . Barriers to the effective management of bipolar disorder: a survey of psychiatrists based in the UK and USA. Bipolar Disord. (2005) 7 Suppl 1:38–42. doi: 10.1111/j.1399-5618.2005.00193.x 15762867

[290] Glauser TA Cerenzia W Wiley S Howson A Thase M . Identifying psychiatrists’ practice patterns when managing depression in patients with bipolar I disorder: a descriptive study to inform education needs. Postgrad Med. (2013) 125:144–53. doi: 10.3810/pgm.2013.01.2606 23391680

[291] Han C Wang SM Lee SJ Patkar AA Masand PS Pae CU . Dilemma for enhancing psychiatrists’ adherence to guideline (evidence)-based practice. Expert Rev Neurother. (2013) 13:751–4. doi: 10.1586/14737175.2013.811196 23898847

[292] Chengappa KR Goodwin GM . Characterizing barriers, challenges and unmet needs in the management of bipolar disorder. Bipolar Disord. (2005) 7 Suppl 1:5–7. doi: 10.1111/j.1399-5618.2005.00188.x 15762863

[293] Bauer MS . A review of quantitative studies of adherence to mental health clinical practice guidelines. Harv Rev Psychiatry. (2002) 10:138–53. doi: 10.1080/10673220216217 12023929

[294] Masand PS Tracy N . Results from an online survey of patient and caregiver perspectives on unmet needs in the treatment of bipolar disorder. Prim Care Companion CNS Disord. (2014) 16. doi: 10.4088/PCC.14m01655 PMC4318674 25664214

[295] Badger TA McNiece C Bonham E Jacobson J Gelenberg AJ . Health outcomes for people with serious mental illness: a case study. Perspect Psychiatr Care. (2003) 39:23–32. doi: 10.1111/j.1744-6163.2003.tb00670.x 12724963

[296] Bauer MS Kirk GF Gavin C Williford WO . Determinants of functional outcome and healthcare costs in bipolar disorder: a high-intensity follow-up study. J Affect Disord. (2001) 65:231–41. doi: 10.1016/s0165-0327(00)00247-0 11511403

[297] Keck PE Jr. McElroy SL Strakowski SM Stanton SP Kizer DL Balistreri TM . Factors associated with pharmacologic noncompliance in patients with mania. J Clin Psychiatry. (1996) 57:292–7. 8666570

[298] Johnson RE McFarland BH . Lithium use and discontinuation in a health maintenance organization. Am J Psychiatry. (1996) 153:993–1000. doi: 10.1176/ajp.153.8.993 8678195

[299] Murru A Pacchiarotti I Amann BL Nivoli AM Vieta E Colom F . Treatment adherence in bipolar I and schizoaffective disorder, bipolar type. J Affect Disord. (2013) 151:1003–8. doi: 10.1016/j.jad.2013.08.026 24099884

[300] Sachs GS . Unmet needs in the assessment and management of bipolar I depression. J Clin Psychiatry. (2013) 74:e11. doi: 10.4088/JCP.12065tx1c 23842019

[301] Fountoulakis KN Young A Yatham L Grunze H Vieta E Blier P . The international college of neuropsychopharmacology (CINP) treatment guidelines for bipolar disorder in adults (CINP-BD-2017), part 1: background and methods of the development of guidelines. Int J Neuropsychopharmacol. (2017) 20:98–120. doi: 10.1093/ijnp/pyw091 27815414 PMC5408969

[302] Hopewell S Clarke M Moher D Wager E Middleton P Altman DG . CONSORT for reporting randomized controlled trials in journal and conference abstracts: explanation and elaboration. PloS Med. (2008) 5:e20. doi: 10.1371/journal.pmed.0050020 18215107 PMC2211558

[303] Liberati A Altman DG Tetzlaff J Mulrow C Gotzsche PC Ioannidis JP . The PRISMA statement for reporting systematic reviews and meta-analyses of studies that evaluate healthcare interventions: explanation and elaboration. Bmj. (2009) 339:b2700. doi: 10.1136/bmj.b2700 19622552 PMC2714672

[304] Moher D Liberati A Tetzlaff J Altman DG Group P . Preferred reporting items for systematic reviews and meta-analyses: the PRISMA statement. BMJ. (2009) 339:b2535. doi: 10.1136/bmj.b2535 19622551 PMC2714657

[305] Moher D Liberati A Tetzlaff J Altman DG Group P . Preferred reporting items for systematic reviews and meta-analyses: the PRISMA statement. PloS Med. (2009) 6:e1000097. doi: 10.1371/journal.pmed.1000097 19621072 PMC2707599

[306] Fountoulakis KN Vieta E . Efficacy and safety of aripiprazole in the treatment of bipolar disorder: a systematic review. Ann Gen Psychiatry. (2009) 8:16. doi: 10.1186/1744-859X-8-16 19635147 PMC2724509

[307] Fountoulakis KN Vieta E Schmidt F . Aripiprazole monotherapy in the treatment of bipolar disorder: a meta-analysis. J Affect Disord. (2011) 133:361–70. doi: 10.1016/j.jad.2010.10.018 21040979

[308] Calabrese JR Bowden CL Sachs GS Ascher JA Monaghan E Rudd GD . A double-blind placebo-controlled study of lamotrigine monotherapy in outpatients with bipolar I depression. Lamictal 602 Study Group. J Clin Psychiatry. (1999) 60:79–88. doi: 10.4088/jcp.v60n0203 10084633

[309] Fletcher SW Spitzer WO . Approach of the Canadian task force to the periodic health examination. Ann Intern Med. (1980) 92:253–4. doi: 10.7326/0003-4819-92-2-253 7352732

[310] Dawes M Summerskill W Glasziou P Cartabellotta A Martin J Hopayian K . Sicily statement on evidence-based practice. BMC Med Educ. (2005) 5:1. doi: 10.1186/1472-6920-5-1 15634359 PMC544887

[311] Richardson WS Wilson MC Nishikawa J Hayward RS . The well-built clinical question: a key to evidence-based decisions. ACP J Club. (1995) 123:A12–13. doi: 10.7326/ACPJC-1995-123-3-A12 7582737

[312] Schlosser RW Koul R Costello J . Asking well-built questions for evidence-based practice in augmentative and alternative communication. J Commun Disord. (2007) 40:225–38. doi: 10.1016/j.jcomdis.2006.06.008 16876187

[313] Rosenberg WM Deeks J Lusher A Snowball R Dooley G Sackett D . Improving searching skills and evidence retrieval. J R Coll Physicians Lond. (1998) 32:557–63. doi: 10.1016/S0035-8819(25)01804-5 PMC9662986 9881313

[314] Horsley T Hyde C Santesso N Parkes J Milne R Stewart R . Teaching critical appraisal skills in healthcare settings. Cochrane Database Syst Rev. (2011) 2011:CD001270. doi: 10.1002/14651858.CD001270.pub2 22071800 PMC7389530

[315] Parkes J Hyde C Deeks J Milne R . Teaching critical appraisal skills in health care settings. Cochrane Database Syst Rev. (2001) 3:CD001270. doi: 10.1002/14651858.CD001270 11686986

[316] Ivers N Jamtvedt G Flottorp S Young JM Odgaard-Jensen J French SD . Audit and feedback: effects on professional practice and healthcare outcomes. Cochrane Database Syst Rev. (2012) 2012:CD000259. doi: 10.1002/14651858.CD000259.pub3 22696318 PMC11338587

[317] Jamtvedt G Young JM Kristoffersen DT O’Brien MA Oxman AD . Audit and feedback: effects on professional practice and health care outcomes. Cochrane Database Syst Rev. (2006) 2:CD000259. doi: 10.1002/14651858.CD000259.pub2 16625533

[318] Jamtvedt G Young JM Kristoffersen DT O’Brien MA Oxman AD . Does telling people what they have been doing change what they do? A systematic review of the effects of audit and feedback. Qual Saf Health Care. (2006) 15:433–6. doi: 10.1136/qshc.2006.018549 PMC2464905 17142594

[319] Jamtvedt G Young JM Kristoffersen DT Thomson O’Brien MA Oxman AD . Audit and feedback: effects on professional practice and health care outcomes. Cochrane Database Syst Rev. (2003) 3:CD000259. doi: 10.1002/14651858.CD000259 12917891

[320] Tonelli MR . In defense of expert opinion. Acad Med. (1999) 74:1187–92. doi: 10.1097/00001888-199911000-00010 10587679

[321] Sherman M Burak K Maroun J Metrakos P Knox JJ Myers RP . Multidisciplinary Canadian consensus recommendations for the management and treatment of hepatocellular carcinoma. Curr Oncol. (2011) 18:228–40. doi: 10.3747/co.v18i5.952 PMC3185900 21980250

[322] U.S. Preventive Services Task Force . Guide to clinical preventive services: report of the U.S. Preventive Services Task Force. DIANE Publishing (1989).

[323] OCEBM Levels of Evidence Working Group . The Oxford 2011 levels of evidence (2015). Available online at: http://www.cebm.net/index.aspx?o=5653 (Accessed March 30, 2025).

[324] Paul C Gallini A Archier E Castela E Devaux S Aractingi S . Evidence-based recommendations on topical treatment and phototherapy of psoriasis: systematic review and expert opinion of a panel of dermatologists. J Eur Acad Dermatol Venereol. (2012) 26 Suppl 3:1–10. doi: 10.1111/j.1468-3083.2012.04518.x 22512675

[325] Lehman AF Steinwachs DM . Translating research into practice: the Schizophrenia Patient Outcomes Research Team (PORT) treatment recommendations. Schizophr Bull. (1998) 24:1–10. doi: 10.1093/oxfordjournals.schbul.a033302 9502542

[326] Grunze H Kasper S Goodwin G Bowden C Baldwin D Licht R . World Federation of Societies of Biological Psychiatry (WFSBP) guidelines for biological treatment of bipolar disorders. Part I: Treatment of bipolar depression. World J Biol Psychiatry. (2002) 3:115–24. doi: 10.3109/15622970209150612 12478876

[327] Grunze H Kasper S Goodwin G Bowden C Baldwin D Licht RW . The world federation of societies of biological psychiatry (WFSBP) guidelines for the biological treatment of bipolar disorders, part II: treatment of mania. World J Biol Psychiatry. (2003) 4:5–13. doi: 10.3109/15622970309167904 12582971

[328] Grunze H Kasper S Goodwin G Bowden C Moller HJ Disorders, W. T. F. o. T. G. f. B . The World Federation of Societies of Biological Psychiatry (WFSBP) guidelines for the biological treatment of bipolar disorders, part III: maintenance treatment. World J Biol Psychiatry. (2004) 5:120–35. doi: 10.1080/15622970410029924 15346536

[329] Guyatt GH Oxman AD Kunz R Vist GE Falck-Ytter Y Schunemann HJ . What is “quality of evidence” and why is it important to clinicians? BMJ. (2008) 336:995–8. doi: 10.1136/bmj.39490.551019.BE PMC2364804 18456631

[330] Jaeschke R Guyatt GH Dellinger P Schunemann H Levy MM Kunz R . Use of GRADE grid to reach decisions on clinical practice guidelines when consensus is elusive. Bmj. (2008) 337:a744. doi: 10.1136/bmj.a744 18669566

[331] Oxman AD Guyatt GH . Guidelines for reading literature reviews. CMAJ. (1988) 138:697–703. 3355948 PMC1267776

[332] Schunemann HJ Fretheim A Oxman AD . Improving the use of research evidence in guideline development: 10. Integrating values and consumer involvement. Health Res Policy Syst. (2006) 4:22. doi: 10.1186/1478-4505-4-22 17147811 PMC1697808

[333] Guyatt G Oxman AD Sultan S Brozek J Glasziou P Alonso-Coello P . GRADE guidelines: 11. Making an overall rating of confidence in effect estimates for a single outcome and for all outcomes. J Clin Epidemiol. (2013) 66:151–7. doi: 10.1016/j.jclinepi.2012.01.006 22542023

[334] Guyatt GH Oxman AD Kunz R Brozek J Alonso-Coello P Rind D . GRADE guidelines 6. Rating the quality of evidence–imprecision. J Clin Epidemiol. (2011) 64:1283–93. doi: 10.1016/j.jclinepi.2011.01.012 21839614

[335] Guyatt GH Oxman AD Kunz R Woodcock J Brozek J Helfand M . GRADE guidelines: 8. Rating the quality of evidence–indirectness. J Clin Epidemiol. (2011) 64:1303–10. doi: 10.1016/j.jclinepi.2011.04.014 21802903

[336] Guyatt GH Oxman AD Kunz R Woodcock J Brozek J Helfand M . GRADE guidelines: 7. Rating the quality of evidence–inconsistency. J Clin Epidemiol. (2011) 64:1294–302. doi: 10.1016/j.jclinepi.2011.03.017 21803546

[337] Guyatt GH Oxman AD Montori V Vist G Kunz R Brozek J . GRADE guidelines: 5. Rating the quality of evidence–publication bias. J Clin Epidemiol. (2011) 64:1277–82. doi: 10.1016/j.jclinepi.2011.01.011 21802904

[338] Guyatt GH Oxman AD Sultan S Glasziou P Akl EA Alonso-Coello P . GRADE guidelines: 9. Rating up the quality of evidence. J Clin Epidemiol. (2011) 64:1311–6. doi: 10.1016/j.jclinepi.2011.06.004 21802902

[339] Guyatt GH Oxman AD Vist G Kunz R Brozek J Alonso-Coello P . GRADE guidelines: 4. Rating the quality of evidence–study limitations (risk of bias). J Clin Epidemiol. (2011) 64:407–15. doi: 10.1016/j.jclinepi.2010.07.017 21247734

[340] Guyatt GH Oxman AD Vist GE Kunz R Falck-Ytter Y Alonso-Coello P . GRADE: an emerging consensus on rating quality of evidence and strength of recommendations. BMJ. (2008) 336:924–6. doi: 10.1136/bmj.39489.470347.AD PMC2335261 18436948

[341] Fountoulakis KN Vieta E Young A Yatham L Grunze H Blier P . The international college of neuropsychopharmacology (CINP) treatment guidelines for bipolar disorder in adults (CINP-BD-2017), part 4: unmet needs in the treatment of bipolar disorder and recommendations for future research. Int J Neuropsychopharmacol. (2017) 20:196–205. doi: 10.1093/ijnp/pyw072 27677983 PMC5408978

[342] Fountoulakis KN Yatham L Grunze H Vieta E Young A Blier P . The international college of neuro-psychopharmacology (CINP) treatment guidelines for bipolar disorder in adults (CINP-BD-2017), part 2: review, grading of the evidence, and a precise algorithm. Int J Neuropsychopharmacol. (2017) 20:121–79. doi: 10.1093/ijnp/pyw100 PMC5409012 27816941

[343] Ghaemi SN Pardo TB Hsu DJ . Strategies for preventing the recurrence of bipolar disorder. J Clin Psychiatry 65 Suppl. (2004) 10:16–23. 15242328

[344] American Psychiatric Association . Diagnostic and Statistical Manual of Mental Disorders 4th Edition, Text Revision, DSM-IV-TR. Washington, DC: American Psychiatric Publishing (2000).

[345] Calabrese JR Goldberg JF Ketter TA Suppes T Frye M White R . Recurrence in bipolar I disorder: a *post hoc* analysis excluding relapses in two double-blind maintenance studies. Biol Psychiatry. (2006) 59:1061–4. doi: 10.1016/j.biopsych.2006.02.034 16769295

[346] Fountoulakis KN . Refractoriness in bipolar disorder: definitions and evidence-based treatment. CNS Neurosci Ther. (2012) 18:227–37. doi: 10.1111/j.1755-5949.2011.00259.x PMC6493614 22070611

[347] Cipriani A Barbui C Rendell J Geddes JR . Clinical and regulatory implications of active run-in phases in long-term studies for bipolar disorder. Acta Psychiatr Scand. (2014) 129:328–42. doi: 10.1111/acps.12223 24289821

[348] Grande I Bernardo M Bobes J Saiz-Ruiz J Alamo C Vieta E . Antipsychotic switching in bipolar disorders: a systematic review. Int J Neuropsychopharmacol. (2014) 17:497–507. doi: 10.1017/S1461145713001168 24139622

[349] Calabrese JR Fava M Garibaldi G Grunze H Krystal AD Laughren T . Methodological approaches and magnitude of the clinical unmet need associated with amotivation in mood disorders. J Affect Disord. (2014) 168:439–51. doi: 10.1016/j.jad.2014.06.056 25113957

[350] Frye MA Prieto ML Bobo WV Kung S Veldic M Alarcon RD . Current landscape, unmet needs, and future directions for treatment of bipolar depression. J Affect Disord. (2014) 169 Suppl 1:S17–23. doi: 10.1016/S0165-0327(14)70005-9 25533910

[351] McElroy SL . Pros and cons of approved therapies for bipolar depression and ongoing unmet needs. J Clin Psychiatry. (2014) 75:e26. doi: 10.4088/JCP.13019tx4c 25373131

[352] Akiskal HS Walker P Puzantian VR King D Rosenthal TL Dranon M . Bipolar outcome in the course of depressive illness. Phenomenologic, familial, and pharmacologic predictors. J Affect Disord. (1983) 5:115–28. doi: 10.1016/0165-0327(83)90004-6 6222091

[353] Weissman MM Prusoff BA Merikangas KR . Is delusional depression related to bipolar disorder? Am J Psychiatry. (1984) 141:892–3. doi: 10.1176/ajp.141.7.892 6731641

[354] Bonnin CM Reinares M Hidalgo-Mazzei D Undurraga J Mur M Saez C . Predictors of functional outcome after a manic episode. J Affect Disord. (2015) 182:121–5. doi: 10.1016/j.jad.2015.04.043 25985381

[355] Thase ME Jonas A Khan A Bowden CL Wu X McQuade RD . Aripiprazole monotherapy in nonpsychotic bipolar I depression: results of 2 randomized, placebo-controlled studies. J Clin Psychopharmacol. (2008) 28:13–20. doi: 10.1097/jcp.0b013e3181618eb4 18204335

[356] Loebel A Cucchiaro J Silva R Kroger H Hsu J Sarma K . Lurasidone monotherapy in the treatment of bipolar I depression: a randomized, double-blind, placebo-controlled study. Am J Psychiatry. (2014) 171:160–8. doi: 10.1176/appi.ajp.2013.13070984 24170180

[357] Vieta E Cruz N . Head to head comparisons as an alternative to placebo-controlled trials. Eur Neuropsychopharmacol. (2012) 22:800–3. doi: 10.1016/j.euroneuro.2011.11.011 22205018

[358] Siddiqui O Hung HM O’Neill R . MMRM vs. LOCF: a comprehensive comparison based on simulation study and 25 NDA datasets. J Biopharm Stat. (2009) 19:227–46. doi: 10.1080/10543400802609797 19212876

[359] Fountoulakis K . Disability and overall burden related with bipolar disorder. In: Fountoulakis K , editor. Bipolar Disorder: An Evidence-Based Guide to Manic Depression. Springer-Verlag Berlin Heidelberg (2015). p. 361–88.

[360] Young AH Rigney U Shaw S Emmas C Thompson JM . Annual cost of managing bipolar disorder to the UK healthcare system. J Affect Disord. (2011) 133:450–6. doi: 10.1016/j.jad.2011.06.016 21737141

[361] Begley CE Annegers JF Swann AC Lewis C Coan S Schnapp WB . The lifetime cost of bipolar disorder in the US: an estimate for new cases in 1998. Pharmacoeconomics. (2001) 19:483–95. doi: 10.2165/00019053-200119050-00004 11465308

[362] McCrone P Dhanasiri S Patel A Knapp M Lawton-Simth S . Paying the price. London: The King’s Fund (2008).

[363] Wyatt RJ Henter I . An economic evaluation of manic-depressive illness–1991. Soc Psychiatry Psychiatr Epidemiol. (1995) 30:213–9. doi: 10.1007/BF00789056 PMC4301427 7482006

[364] Runge C Grunze H . Annual costs of bipolar disorders in Germany. Nervenarzt. (2004) 75:896–903. doi: 10.1007/s00115-004-1691-x 14999464

[365] Ekman M Granstrom O Omerov S Jacob J Landen M . The societal cost of bipolar disorder in Sweden. Soc Psychiatry Psychiatr Epidemiol. (2013) 48:1601–10. doi: 10.1007/s00127-013-0724-9 23754681

[366] Fisher LJ Goldney RD Dal Grande E Taylor AW Hawthorne G . Bipolar disorders in Australia. A population-based study of excess costs. Soc Psychiatry Psychiatr Epidemiol. (2007) 42:105–9. doi: 10.1007/s00127-006-0133-4 17080320

[367] Hakkaart-van Roijen L Hoeijenbos MB Regeer EJ ten Have M Nolen WA Veraart CP . The societal costs and quality of life of patients suffering from bipolar disorder in the Netherlands. Acta Psychiatr Scand. (2004) 110:383–92. doi: 10.1111/j.1600-0447.2004.00403.x 15458562

[368] Hidalgo-Mazzei D Undurraga J Reinares M Bonnin Cdel M Saez C Mur M . The real world cost and health resource utilization associated to manic episodes: The MANACOR study. Rev Psiquiatr Salud Ment. (2015) 8:55–64. doi: 10.1016/j.rpsm.2015.01.003 25752959

